# 3D Membrane Microstructures for Increased Efficiency in Blood‐Gas Transfer

**DOI:** 10.1002/advs.202512302

**Published:** 2025-11-07

**Authors:** Kai P. Barbian, F. Neuhaus, L. T. Hirschwald, J. Linkhorst, M. Wessling, B. Wiegmann, J. M. Focke, U. Steinseifer, M. Neidlin, S. V. Jansen

**Affiliations:** ^1^ Department of Cardiovascular Engineering Institute of Applied Medical Engineering Medical Faculty RWTH Aachen University Forckenbeckstr. 55 52074 Aachen Germany; ^2^ Chemical Process Engineering (AVT.CVT) RWTH Aachen University Forckenbeckstr. 51 52074 Aachen Germany; ^3^ Process Engineering of Electrochemical Systems Technical University of Darmstadt Otto‐Berndt‐Str. 2 64287 Darmstadt Germany; ^4^ DWI, Leibniz‐Institute for Interactive Materials Forckenbeckstr. 50 52074 Aachen Germany; ^5^ Department for Cardiothoracic Transplantation and Vascular Surgery Hannover Medical School Carl‐Neuberg‐Straße 1 30625 Hannover Germany; ^6^ Implant Research and Development (NIFE) Lower Saxony Center for Biomedical Engineering Stadtfelddamm 34 30625 Hannover Germany; ^7^ German Center for Lung Research (DZL) Carl‐Neuberg‐Straße 1 30625 Hannover Germany

**Keywords:** blood contactors, ECMO, mass transfer, membrane oxygenators, microstructures, TPMS

## Abstract

Treating severe lung diseases with extracorporeal membrane oxygenation (ECMO) is often accompanied by serious complications, restricting applicability and adversely affecting outcomes. Membrane oxygenators, based on hollow fiber membranes (HFM), are a central ECMO component. Here, current designs are reaching their limits regarding hemocompatibility and gas transfer efficiency. Three‐dimensional structures, based on triply periodic minimal surfaces, can provide superior hemodynamics. However, previous 3D membrane structures don't provide the required channel scale and gas transfer performance. In this study, we have fabricated 3D microstructures and tested them in vitro against state‐of‐the‐art HFM. The 3D structure provided gas transfer coefficients within 50 and 45% of the HFM's value for oxygen and carbon dioxide, respectively. Specific pressure drops within the 3D structure are more than ten times lower than in the HFM. Combining these aspects, the 3D structure showed 51and 33% higher mass‐transfer efficiency for oxygen and carbon dioxide, respectively. By further reducing channel scale, the 3D structure can help to miniaturize oxygenators while maintaining efficiency. Thereby, the invasiveness of ECMO could be reduced to improve outcomes. Our approach for the design and manufacturing of 3D membrane microstructures can be transferred to other mass‐transfer applications like bioreactors or microfluidic cell culture.

## Graphical abstract

Oxygenators for extracorporeal membrane oxygenation are based on hollow fiber membranes. They are associated with many complications, such as thrombosis or systemic inflammation during the therapy. By implementing 3D membrane microstructures for oxygenators, the transfer efficiency of oxygen and carbon dioxide is improved, which can lead to miniaturized, more hemocompatible systems in the future for reduced complication rates.

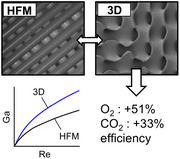

## 1 Introduction

Severe chronic lung diseases, like chronic obstructive pulmonary disease, are the third most common cause of death worldwide. The total number of people suffering from this disease is estimated to be around 300 million, with an upward trend, and is predicted to reach 600 million by 2050.^[^ ^1^, ^2^ ^]^ Despite hospital treatment, around 15% of all patients die after a so‐called exacerbation, a sudden worsening of the disease.^[^ ^3^ ^]^ In addition, patients suffer from a low quality of life.^[^ ^4^ ^]^ The corresponding long hospital stays are also a financial burden on the healthcare system.^[^ ^5^ ^]^ Severe cases of chronic lung diseases are usually treated with mechanical ventilation. If this treatment option is no longer sufficient, extracorporeal membrane oxygenation (ECMO) is the only remaining therapy apart from transplantation. Here, the patient's blood passes through an oxygenator, outside of the human body, where it is enriched with oxygen while carbon dioxide is removed. State‐of‐the‐art oxygenators are based exclusively on the concept of hollow fiber membranes (HFM). This membrane concept has been used for over 50 years with little potential for further innovation.^[^ ^6^ ^]^ Here, gas flows through the inner fiber lumina while blood flows through the interstitial spaces between the fibers. However, this inherently unphysiological flow within these devices is a cause of both stagnation zones and uneven blood distribution, where high shear rates further trigger blood activation.^[^ ^7^ ^]^ This not only wastes potential gas‐exchange capacity but also promotes thrombus formation.^[^ ^8^, ^9^ ^]^ Clinically, thrombus formation is countered by high‐dose systemic anticoagulation, which in turn can lead to internal bleeding. Thrombus formation inside the oxygenator limits its use to a few days on average.^[^ ^10^, ^11^, ^12^ ^]^ The pressure drop of the oxygenator must be compensated for by an extracorporeal blood pump. Here, the occurrence of high‐pressure losses in the oxygenator is associated with increased mechanical blood damage (hemolysis) inside the pump.^[^ ^13^ ^]^ At the same time, the extensive foreign surface area in contact with blood drives systemic inflammatory responses. With ECMO mortality remaining as high as 50%, a next‐generation membrane technology must eliminate stagnation zones, excessive shear stress, and substantially increase gas transfer efficiency in order to reduce surface area and thus achieve higher biocompatibility.^[^ ^14^, ^15^ ^]^

One approach for the innovation of ECMO therapy is the development of microfluidic artificial lungs (MAL). Here, small capillary‐like flow channels mimic physiological conditions and increase the area available for gas transfer.^[^ ^16^, ^17^ ^]^ Besides that, 3D membrane structures, based on triply periodic minimal surfaces (TPMS), have been proposed to provide several advantages over conventional HFM technology. Using the 3D structure, micro‐stagnation zones within the blood flow are reduced, decreasing the risk of thrombus formation.^[^ ^18^ ^]^ Moreover, gas transfer efficiency can be increased by passive blood mixing effects within the 3D structure.^[^ ^19^, ^20^, ^21^ ^]^ However, these findings resulted from similarity theory and 3D structures with channel scales and membrane thicknesses of six to nine times the feature sizes of clinically used HFM. Thus, the final proof on whether 3D TPMS membrane structures are truly beneficial as an alternative for HFM is missing. Three‐dimensional membrane structures, based on TPMS, that provide the channel dimensions required to be truly comparable and competitive with clinically used HFM, have never been manufactured and tested before. The biggest challenge here is the manufacturing of gas‐permeable membrane structures of this complex geometry with the required small dimensions. To this date, no commercial manufacturing method is available to build these 3D membrane structures directly and is suitable for blood gas transfer.

**Figure 1:**
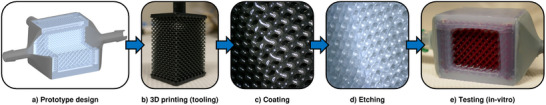
Overview of different steps conducted for the performance evaluation of the 3D membrane structure. a) Design of the oxygenator prototype, b) 3D printing of the tool geometry, c) coating of the tool geometry to form the gas‐permeable membrane, d) etching of the 3D printing material, e) and in vitro measurement of gas transfer performance and pressure drop.

To overcome these challenges, we developed a new, hybrid manufacturing process that combines high‐resolution 3D printing with dip‐spin coating for the creation of a thin membrane (cf. **Figure** **1**). Thereby, a gas‐permeable, multi‐scale membrane structure can be created with membrane thicknesses in the scale of micrometers inside a coherent structure measuring tens of centimeters. We designed miniaturized oxygenators based on the 3D membrane structure, and fabricated them with our developed process. Afterward, we evaluated the structures in terms of overall fabrication quality, feature sizes, and surface smoothness. Finally, we performed gas transfer performance testing on the 3D membrane modules. This test was conducted in direct comparison with prototypes of HFM oxygenators with similar surface area using porcine blood. Thereby, the functionality of 3D TPMS membrane structures in a clinically realistic scale is evaluated for the first time.

## 2 Results

### 2.1 Fabrication Quality

**Figure 2:**
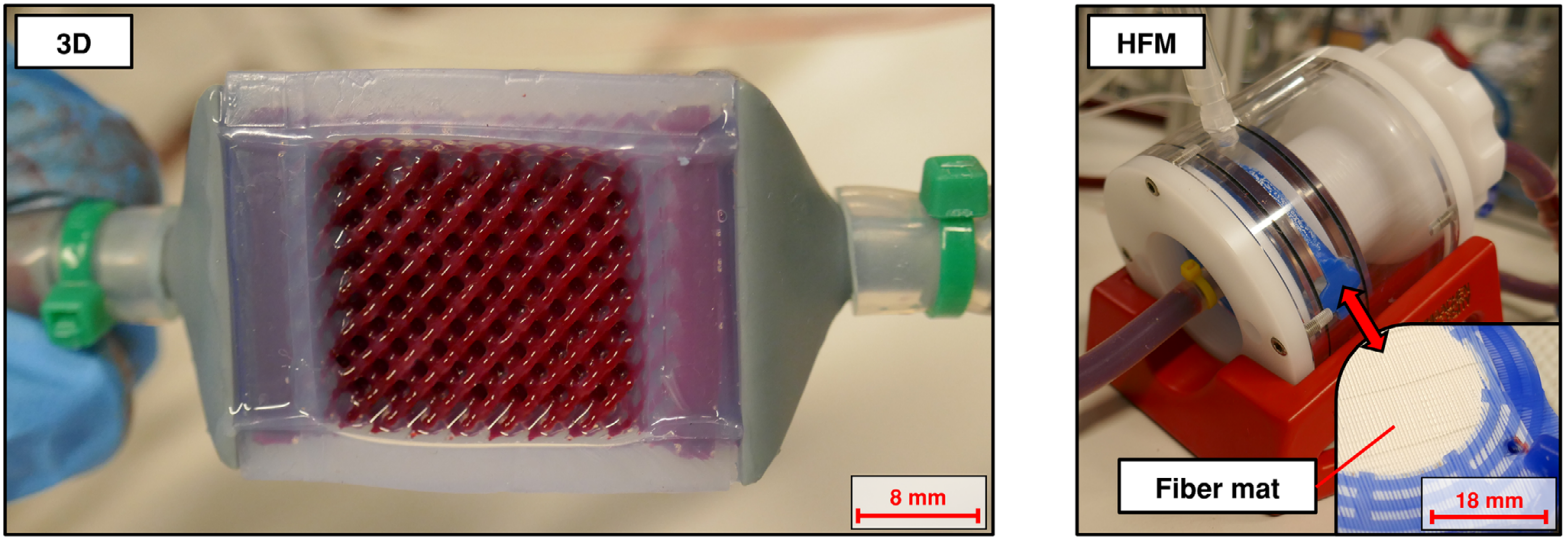
Images of the 3D membrane oxygenator (left) and HFM oxygenator (right) prototypes during in vitro testing.

Our proposed manufacturing approach enabled the fabrication of micro‐scale 3D membrane structures for use in laboratory‐scale oxygenator prototypes. The manufactured modules were 24 mm in height and width and 28 mm in length (blood flow direction), featuring a blood contact surface area of 14 745 mm^2^. The blood priming volume inside the membrane section of the module was 4.8 mL (calculated). The distribution end caps at the blood inlet and outlet featured a priming volume of 3.4 mL each. This adds up to a total priming volume of 11.6 mL. However, the end caps were not optimized to achieve a small priming volume, which could be reduced significantly in further design iterations. The HFM oxygenator prototypes featured a priming volume of 2.3 mL, where the membrane bundle accounted for 1.3 mL and the end caps for 0.5 mL, each. For TPMS geometries, the unit cell size is a characteristic parameter that defines the length of the smallest periodic sub‐unit of the structure in each coordinate axis. The modules that were used for the in vitro testing were built with a unit cell size of 3 mm. All six manufactured modules did not show any signs of leakage during the testing of watertightness and during the performance tests with blood, which were carried out with five of the final prototypes (cf. **Figure** **2**). Moreover, full channel patency was observed during these tests. The manufactured modules with a 1 mm unit cell size showed defects that did not make them suitable for in vitro testing. Still, large areas of these modules proved to be defect‐free (cf. Figure 4). During the casting process of the outer sealing, silicone was drawn into the gas flow channels of the modules due to capillary effects (cf. Figure 2; Figure S1, Supporting Information). Therefore, the active membrane area available for gas transfer was reduced.

**Figure 3:**
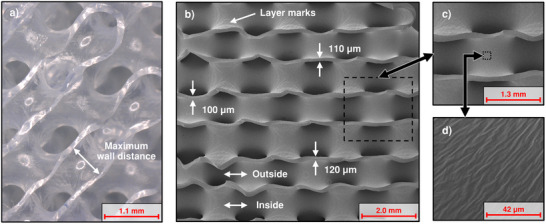
Microscopic images of a cross‐section cut through a 3 mm unit cell‐sized membrane module. a) Light microscopy image, b) SEM image with measurements of membrane thickness and indication of inside and outside of the membrane structure during the dip‐spin coating process, c) close‐up of a section of b), d) close‐up of a section of c) revealing the surface structure of the smooth membrane side.

**Figure 4:**
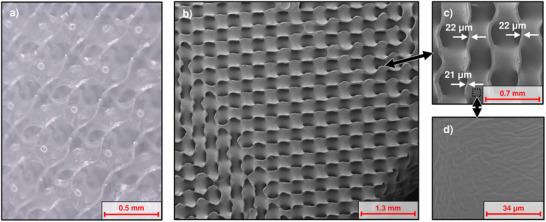
Microscopic images of a cross‐section cut through a 1 mm unit cell size membrane module. a) Light microscopy image, b) SEM image showing the 3D structure, c) close‐up of a section of b) with measurements of membrane thickness, d) close‐up of a section of c) revealing the surface structure of the smooth membrane side.

Light microscopy and scanning electron microscopy (SEM) images of a slice‐section cut through the prototypes are shown in **Figures** **3** and 4 for the unit cell sizes of 3 and 1 mm, respectively. The membrane thickness can be evaluated at the points where the cut through the membrane is orthogonal to its surface (cf. Figure 3b). Measurements of the membrane thickness showed a distribution between 96 and 124 µm with an average of 112 µm. Moreover, no general tendency of membrane thickness variation in different locations (e.g., inside/outside) was observed (cf. Figure S2, Supporting Information). The surface structure of two neighboring channels differs in terms of their roughness. On the side of the membrane that was in contact with the tool body during coating (“inside”), the surface structure of the 3D prints with the typical stair‐stepping effects in the scale of 20 µm can be clearly recognized (cf. Figure 3b). The other side of the membrane has a much smoother surface (“outside”). Only at very high magnification, small wrinkles in the order of a few micrometers in separation can be identified (cf. Figure 3d). These wrinkles appear on the inside and the outside surface of the membrane. Potential causes could be the sputtering process for the SEM, silicone run off during the centrifugation process, or solvent evaporation or sorption effects.^[^ ^22^ ^]^ However, we could not clearly identify the effect that introduced these wrinkles.

The prototypes with a 1 mm unit cell size also showed a high production quality at this significantly smaller scale. This confirmed that the proposed manufacturing process is capable of accurately molding channel widths of approximately 350 µm. In some areas of the prototypes, blockages within the channels were observed, which occur when the silicone is not sufficiently removed from the voids during centrifugation. Nevertheless, most areas of the membrane are free from defects (cf. **Figure** **4**b). Homogeneous membrane thicknesses of down to 21 µm were produced within these modules (cf. Figure 4c). Here, the same surface structure as in the 3 mm prototypes can be observed (cf. Figure 4d).

**Table** **1** gives a comparison of the membrane types and characteristics of both the HFM and the 3D membrane structure. The membrane within the 3D structure was built as a dense layer of silicone, while the HFM was a commercially available type made of polymethylpentene (PMP) with solid outer layers and a microporous inner structure, typically used for oxygenators. By using a similar cross‐sectional area of the membrane modules, the blood flow velocities were matched. To also make the membrane surface areas similar, the lengths of the modules had to be adapted. Here, the lengths were adjusted in increments of HFM layer mat thickness or TPMS unit cell size. Because of the drawn‐in silicone inside the gas side channels at the outer sealing, the active membrane surface area that is available for gas transfer was corrected for the 3D structure (cf. Figure 2; Figure S1, Supporting Information). The characteristic dimension was chosen to be the maximum normal wall distance for the 3D structure (cf. Figure 3) and the fiber diameter for the HFM.

**Table 1:** Specifications of membrane types and modules of the 3D structure and HFM prototypes used for in vitro testing.

	Membrane type	Material	Membrane thickness in µm	Surface area in mm^2^	Cross‐sectional area in mm^2^	Module length in mm	Char. dimension in mm
HFM	Hybrid (microporous/dense)	PMP	90 ± 10/9 (total/dense)	15 822	490.87	6	0.38
3D (3 mm)	Dense	Silicone	110 ± 14	14 745/8294 (total/corrected)	576	28	1.06

### 2.2 Gas Transfer Efficiency

**Figure 5:**
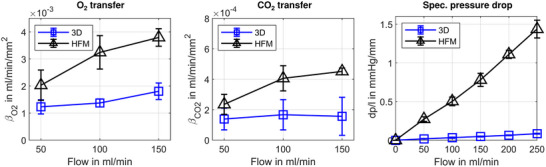
Gas transfer coefficients for oxygen (left) and carbon dioxide (centre), and specific pressure drops (right) from in vitro‐testing results for the 3D membrane structure (“3D”, blue line, square markers) and HFM (“HFM”, black line, triangle markers), presented as mean values and standard deviation for *n* = 5 prototypes.

In **Figure** **5**, a comparison of the oxygen and carbon dioxide transfer performance is provided in terms of the transfer coefficient, *β*_i_, for different flow rates. Here, the total transferred gas flow at standard conditions is referenced to the active membrane surface area and a representative mass species concentration gradient that drives mass transport between sweep gas and blood. Overall, the 3D membrane structure has a significantly lower oxygen transfer at the flow rates of 50, 100, and 150 mL min^−1^ with 61, 58, and 48% of the HFM's oxygen transfer coefficient, respectively. With increasing blood flow, the difference between the structure's oxygen transfer coefficients increases. Noticeably, the full gas transfer potential of the 3D membrane, indicated by the asymptotic progression of the gas transfer coefficient, is reached at lower flow rates. The HFM requires a higher flow rate before the asymptotic plateau of the maximum gas transfer coefficient is achieved.^[^ ^23^ ^]^ Concerning carbon dioxide transfer, a very similar characteristic can be observed with slightly different absolute values. Here, the gas transfer coefficient of the 3D structure is 41 to 65% of the HFM's value. Looking at the specific pressure drop, the absolute pressure loss divided by the module length (cf. Figure 5), a significantly lower value can be observed for the 3D membrane. The specific pressure loss of the 3D membrane of 0.09 mmHg mm^−1^ is more than one order of magnitude lower than that of the HFM with 1.44 mmHg mm^−1^ at a 250 mL min^−1^ blood flow rate. In general, a linear increase in pressure drop with flow rate is observed for both structures. In Figure S3 (Supporting Information), the respective mass transfer rates per membrane area are provided additionally. The differences in gas transfer coefficients and specific pressure drops between the two structures all showed statistical significance, apart from the carbon dioxide transfer coefficient at 50 mL min^−1^ flow rate. Here, a *p*‐value of 0.057 was calculated.

**Figure 6:**
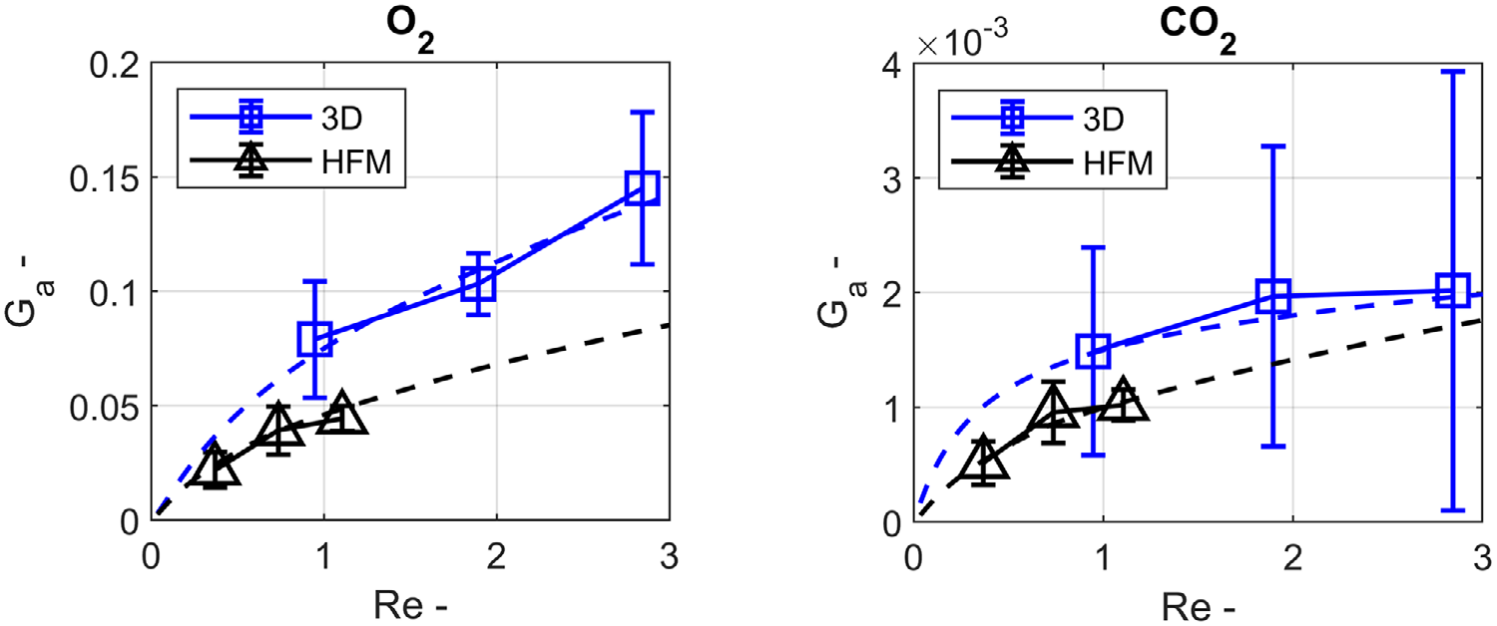
Area goodness factors for oxygen (left) and carbon dioxide (right) calculated from in vitro testing results for the 3D membrane structure (“3D”, blue line, and square markers) and HFM (“HFM”, black line, triangle markers), presented as mean values and standard deviation for *n* = 5 prototypes. Empirical fits are given by the blue dashed lines for the 3D structure and the black dashed lines for the HFM.

To put these two characteristics of gas transfer and pressure drop into relation, area goodness factors (G_a_) were calculated for both structures.^[^ ^24^ ^]^ This factor puts dimensionless mass transfer in relation to dimensionless pressure loss on the available membrane surface area (cf. **Figure** **6**). Moreover, G_a_ is plotted against the Reynolds number for comparing both structures, independent of geometrical scale. Thereby, only the influence of the membrane structure's shape on gas transfer efficiency is compared. The curve of G_a_ over Re is unique for each surface chape and remains the same for different TPMS unit cell sizes (cf. Figure S4, Supporting Information). Here, the overall efficiency of oxygen transfer for the 3D membrane is between 43 and 55% higher across the range of investigated Reynolds numbers. Concerning carbon dioxide transfer, the efficiency of the 3D membrane structure is between 6 and 76% higher than that of the HFM in the investigated range. However, the standard deviation of the G_a_ for carbon dioxide, which was calculated from the measurement data, is high. Therefore, the empirical fit is less reliable. In summary, these results show that the 3D structure can yield higher gas transfer rates for oxygen and carbon dioxide using the same membrane surface area at the same pressure drop as HFM. Raw measurement data of blood samples and pressure drop are provided in Tables S1 and S2 (Supporting Information).

After the in vitro experiments, the prototypes were rinsed with distilled water and inspected for blood residues. No signs of clotting or blood residues were found in any of the prototypes. In **Table** **2**, a performance comparison of the prototypes from this study to different MAL and a clinically used HFM oxygenator for neonatal applications is provided. Here, the area‐specific oxygen and carbon dioxide transfer rates are shown along with the corresponding blood flow rate, the characteristic dimension that describes the geometric scale of the smallest features, the pressure drop, and priming volume.

**Table 2:** Comparison of performance characteristics between the prototypes from this study, different microfluidic artificial lungs, and a commercial HFM oxygenator (“Quadrox‐I Neonatal”).

Source	O_2_ transfer in mL min^−1^ m^−2^	CO_2_ transfer in mL min^−1^ m^−2^	Sweep gas to blood flow ratio	Blood flow in mL min^−1^	Char. dimension in mm	Pressure drop in mmHg	Priming volume in mL
Isenberg et al.^[^ ^17^ ^]^	192	NA	4:1	750	0.24	51.0	117.6^a)^
Fleck et al.^[^ ^25^ ^]^	78	668	16:1	6	0.22	1.6	0.5
Lachaux et al.^[^ ^26^ ^]^	431	651	1:2	45	0.14	69.0	0.8
Quadrox‐i Neonatal^[^ ^27^ ^]^	237	192	1:1	1500	0.38	42.0	40.0
HFM (this study)	203	206	10:1	150	0.38	4.7	2.3
3D (3 mm; this study)	124	75	10:1	150	1.06	1.3	11.6

^a)^ Calculated value, not directly provided

In relation to the published, best‐performing MAL, the 3D structure shows a lower area‐specific oxygen and carbon dioxide transfer. The highest oxygen transfer rate was reported by Lachaux et al.^[^ ^26^ ^]^ Fleck et al. have reported the highest area‐specific carbon dioxide transfer.^[^ ^25^ ^]^ In our study, the oxygen saturation, corrected for human blood, increased from 74% at the inlet to 81% at the outlet on average at a 150 mL min^−1^ flow rate.^[^ ^28^ ^]^ The reported values of area‐specific gas transfer rates of some referenced microfluidic devices are higher than those of the HFM. However, their pressure drops in relation to flow rate are also about one order of magnitude higher. Beyond that, the pressure drop of the prototypes with 3D structure is more than three times lower than that of the HFM prototype from our study. The performance values (area‐specific oxygen transfer, pressure drop in relation to flow rate) of the HFM from our measurements were similar to those reported for clinical use of commercial HFM oxygenators. However, the carbon dioxide transfer in the clinical study of Melchior et al. was slightly lower than that in our measurements due to the utilized 1:1 sweep gas to blood flow ratio, which was 10:1 in our study.^[^ ^27^ ^]^ In summary, the 3D structure shows lower area‐specific gas transfer rates than HFM and the mentioned MAL, but the lowest pressure drops in relation to blood flow. Still, the characteristic dimension of the 3D structure with a 3 mm unit cell size is 2.8 times the HFM scale. As shown in Figure 6, a higher gas transfer rate per membrane surface area can be achieved with a smaller scaled 3D structure at the same or less pressure drop in comparison to the HFM.

Many MAL membranes are built from polydimethylsiloxane (PDMS). Measurements of permeability and diffusivity of the different membrane material samples, PDMS and the used silicone for the TPMS membrane, showed no clear alteration of their permeation characteristics due to the etching process. While there was no such effect observed for diffusivity, PDMS (Sylgard 184) showed a higher permeability for oxygen and carbon dioxide than the membrane material in this study. However, PDMS has a lower mechanical strength. The results of diffusivity and permeability measurements are given in Table S3 (Supporting Information).

## 3 Discussion

3D membrane structures, based on TPMS, have been proposed to provide increased energy transfer efficiency for several applications.^[^ ^29^ ^]^ This can be especially useful for blood gas transfer in the case of ECMO.^[^ ^19^, ^20^ ^]^ However, 3D membrane structures that come close to the clinical gold standard, oxygenators based on HFM, in terms of channel dimensions and performance, have not been built and tested to date. This work presents the manufacturing and testing of the first fully functional oxygenator prototypes with 3D TPMS membrane structures with a clinically relevant channel scale. Only channel sizes below 1 mm can yield the required density of membrane area per priming volume and small gas diffusion distances within the blood that are suitable for clinical use. Directly compared to the conventional HFM, our 3D structure yields a higher mass transfer efficiency for both oxygen and carbon dioxide with respect to hydraulic losses. This efficiency is defined by the area goodness factor (cf. Figure 6). Concerning the manufacturing of the 3D membrane structures, we have developed a process that yields channel sizes and membrane thicknesses in micrometer dimensions. This process consists of the additive manufacturing of a dissolvable tool geometry, on top of which the membrane is formed using dip‐spin coating. Like in semiconductor chip manufacturing, only the 3D membrane structure is preserved after etching away the sacrificial tool geometry. With this process, we manufactured and tested five 3D membrane oxygenators in in vitro blood trials comparing their performance directly to HFM oxygenators of a similar scale. A high overall manufacturing quality of the 3D membrane structures was observed with good shape accuracy, homogeneous membrane thicknesses, and a high surface smoothness. The 3D membrane structure showed smaller gas transfer coefficients than the HFM. Still, its performance was in the same range with oxygen transfer coefficients being within 48 and 61% of the HFM's value. However, the pressure drop inside the 3D structure was significantly lower. This adds up to an average 51 and 33% higher overall transfer efficiency of the 3D structure for oxygen and carbon dioxide transfer, respectively. Here, efficiency was defined by the area goodness factor.

Some studies on the application of TPMS structures for oxygenators have been carried out before. Hesselmann et al. have used sacrificial molds for casting 3D membranes and HFM structures to compare their gas transfer efficiency.^[^ ^19^ ^]^ They also conducted hydraulic testing with blood and reported a 35% increase in oxygen transfer for the Schwarz Diamond TPMS structure and a higher pressure drop in comparison to HFM at the same scale.^[^ ^30^ ^]^ In their study, both the 3D and the HFM structures were upscaled using similarity theory. Moreover, larger membrane thicknesses of 800 µm were present, which increased gas diffusion resistance. When the diffusion resistance is low, like in the real application of HFM, the influence of the structure's geometry on blood flow patterns and total gas transfer rates becomes much more dominant. Another approach for the innovation of ECMO therapy is MAL‐technology. In comparison to the published, best‐performing microfluidic devices, our 3D structure yields an up to 3.5 times lower area‐specific oxygen transfer rate. However, the pressure drop in our device was more than 50 times lower than in some of these devices.^[^ ^26^ ^]^ Moreover, the area‐specific gas transfer rates of MAL are commonly normalized to their active membrane surface area, which is only one‐fourth or one‐half of the total channel wall surface. This aspect is a consequence of the manufacturing limitations of soft lithography. However, the biocompatibility of artificial lungs is impacted by the whole foreign surface area in contact with blood. Using the 3D structure, the whole blood contacting surface area is available for gas transfer, apart from the outer sealing. Directly comparing the raw measurement data of HFM and 3D TPMS prototypes (cf. Table 2), the former performs better regarding gas transfer and priming volume. Smaller wall distances and membrane thicknesses would both increase gas transfer rates by reducing gas diffusion distances inside the blood and lowering the gas transfer resistance of the membrane, respectively. This is because the 3D TPMS prototypes feature wall distances of the blood flow channels and membrane diffusion resistances that are higher than those of HFM. The membrane thickness of our prototypes was 110 µm on average, which is higher than the thickness of most MAL, featuring a membrane thickness between 15 and 80 µm.^[^ ^17^, ^25^, ^26^ ^]^ A larger membrane thickness introduces additional diffusion resistance, reducing gas transfer rates, especially in comparison to the microporous HFM. For HFM, the gas species diffusion resistance through the membrane is typically neglected because it is far lower than the diffusion resistance of the gas species inside the blood.^[^ ^31^ ^]^ As indicated by the area goodness factor (cf. Figure 6), even with this higher membrane diffusion resistance, the 3D structure is superior for use in oxygenators once it is manufactured in a small enough channel scale. Additionally, a smaller wall distance would also increase surface area density per volume for the 3D structure and thus, reduce priming volume when surface area is kept constant.^[^ ^32^ ^]^

Still, we have proven the feasibility of manufacturing 3D TPMS structures with membrane thicknesses down to 20 µm in our 1 mm unit cell size prototypes, which would also result in a very low diffusion resistance of gas through the membrane.

In general, higher area‐specific gas transfer rates are also achieved with decreasing flow channel dimensions. Thereby, the diffusion distances for gas species are reduced within the blood.^[^ ^33^ ^]^ Fleck et al. demonstrated the feasibility of creating a MAL with additive manufacturing directly.^[^ ^25^ ^]^ Their prototype featured channel heights of 200 µm, with two channel walls available for gas transfer. They achieved the highest area‐specific carbon dioxide transfer reported to date. Moreover, moderately higher pressure drops in relation to flow rate were reported, compared to HFM. Despite the design freedom of additive manufacturing, 2D channel geometries were used in their study, and difficulties with channel patency were reported. The direct 3D printing of TPMS membrane structures using stereolithography may be more challenging because of the membrane surface curvature that would lead to alternating membrane orientations in relation to the build direction. In layer‐based 3D printing processes, a part commonly possesses the highest mechanical strength in a perpendicular orientation to the build direction. Our hybrid approach using additive manufacturing and dip‐spin coating reduces the requirements for 3D printing accuracy, as the flow channel geometry is printed instead of the thin membrane wall itself. This allows easier scaling to larger overall dimensions. Moreover, different, already established membrane materials could be implemented into our manufacturing process. This could include materials that form a microporous structure, which can further reduce gas transfer resistance and increase structural strength.

Apart from higher gas transfer efficiencies, TPMS structures offer the potential to reduce thrombosis risk due to the microscopic flow path design. In comparison to HFM, a much smaller variation in shear stress magnitude is observed on the membrane wall (cf. Figure S5, Supporting Information). With the HFM structure, a much broader spectrum of high and low shear stresses can be seen. Supraphysiological shear stress values (>3.6 Pa) could lead to activated platelets that can settle in regions of low shear stress (<< 1 Pa), which are associated with flow stagnation.^[^ ^34^, ^35^, ^36^, ^37^ ^]^ At the investigated flow rates of 150 mL min^−1^, supraphysiologic shear stress values are observed in numerical simulations for the HFM structure at some locations, but not in the 1 and 3 mm TPMS structures. By setting the TPMS unit cell size, the shear stress inside the blood can be adjusted.^[^ ^38^ ^]^

Although we have come close to the characteristic HFM dimension of 380 µm, we have not yet reached its microscopic feature scale for TPMS, yet. They featured a channel width of 1.06 mm, which increases the gas diffusion distance within the blood (cf. Table 2). But with the 1 mm TPMS unit cell size membrane modules, we have demonstrated the manufacturing feasibility of such small channels. Still, these modules showed defects like channel blockages and leakages, which made them inappropriate for in vitro testing. An optimization of the manufacturing parameters to create fully functional 1 mm unit cell size membrane modules is feasible by further tuning the viscosity of the liquid coating solution, the centrifuge rotational speed, and the number of coating layers. Within our testing, the oxygen saturation at the outlet was 81% on average (corrected for human blood^[^ ^28^ ^]^) at the highest flow rate. Our prototypes would be able to sustain physiological conditions (minimum 95% saturation) at a flow rate of approximately 30 mL min^−1^, but not at the highest flow that was investigated. The superiority of the 3D membrane structure over the HFM was proven using the metric of the area goodness factor. This quantity is based on the dimensionless description of gas transfer and pressure loss. For idealized scenarios, like laminar pipe flow with constant fluid properties (viscosity, gas diffusion coefficients) and fixed Reynolds number, the area goodness factor is completely independent of channel scale and only a function of the surface shape. In our study, we investigated the gas transfer efficiency using blood, which has a non‐Newtonian behavior and nonconstant gas species diffusion characteristics due to dissociation effects. In this case, the property of scale independence of the area goodness factor cannot be rigorously proven. However, we have carried out numerical simulations of mass transfer in blood inside TPMS structures that also show a strong independence of the area goodness factor on geometric scale (cf. Figure S4, Supporting Information).^[^ ^32^ ^]^ Moreover, the numeric value of the area goodness factor is dependent on the choice of reference dimension. A change in the reference dimension value would lead to a minor change in the area goodness factor value. This factor was originally reported in heat transfer literature.^[^ ^24^ ^]^ However, because of the analogy between heat and mass transfer, we propose the use of the area goodness factor to compare different surface shapes for blood gas transfer. Mass transfer is always coupled with momentum transfer (pressure loss).^[^ ^23^ ^]^ This factor describes how efficient mass transfer is happening in comparison to momentum transfer, independent of channel size. Absolute values of gas transfer and pressure drop will be defined by a specific device design (e.g., blood flow channel size, membrane surface area, cross‐section, device length, etc.). For two equal designs, a structure with a superior area goodness factor will always yield a higher mass transfer at the same or a lower pressure drop.^[^ ^24^ ^]^

The freestanding 3D membrane structure must also provide the necessary mechanical strength to withstand operating loads. For the 3 mm unit cell size modules, which feature a membrane thickness of 110 µm on average, a small expansion of the prototype was observed under increasing blood pressure. But no ruptures or leakages were observed. However, a lower membrane thickness, like in the 1 mm unit cell modules, would lead to less mechanical strength and thus a higher deformation under pressure. In general, a freestanding 3D membrane structure would need to withstand a higher mechanical load than the channels of current MAL, which are commonly supported by a structural backbone. A significant deformation of the membrane structure was observed from 100 mmHg blood side pressure onward (cf. Figure S6, Supporting Information). A leakage only occurred at 240 mmHg and above. There is no single guideline for pressure values in ECMO. However, 200 to 300 mmHg is generally considered the upper safe limit to prevent patient complications.^[^ ^39^, ^40^, ^41^ ^]^ In their current form, the prototypes could withstand 200 mmHg of pressure. Nevertheless, there is not enough of a safety margin for safe use in the clinic. Moreover, the gas channel size is reduced by blood side expansion. The gas channels stayed open even with 250 mmHg of blood side pressure. Still, gas side flow resistance could be increased with increasing deformation under blood pressure. By using different membrane materials or composites (using micro‐fibers/micro‐spheres) or by modulating the gas side pressure to be equal to the blood side pressure, these issues could be mitigated in the future.

## 4 Conclusion

In summary, we have provided the first 3D TPMS membrane oxygenators with clinically relevant membrane thicknesses and channel sizes. We have shown that this technology outperforms the gold standard of HFM with respect to mass transfer efficiency. Although in the same range, the gas transfer per membrane surface area of the 3D TPMS membrane oxygenators still lags behind the HFM and other MAL. However, the much lower pressure drop of the 3D TPMS structures offers many opportunities for further improvement, such as their manufacturing with smaller unit cell sizes. We have proven the feasibility of manufacturing these structures in the required dimensions using our proposed method. By establishing smaller channel dimensions, a membrane technology can be implemented that provides significantly higher gas transfer rates than HFM over the same membrane surface area at an equal or lower pressure drop. For clinical application, this would mean reduced priming volume, reduced pressure drops, and less foreign surface area in oxygenators to reduce complication rates and improve therapy outcomes. Moreover, 3D membrane structures, based on TPMS, are expected to provide increased hemocompatibility in terms of thrombosis risk. Our approach for the design and manufacturing of modules with a gas‐permeable, 3D membrane structure could also be adapted to other technical and biomedical areas, where an efficiency increase in mass transfer applications is beneficial, like in membrane contactors, bioreactors, and microfluidic cell culture.

## 5 Experimental Section

### Design

**Figure 7:**
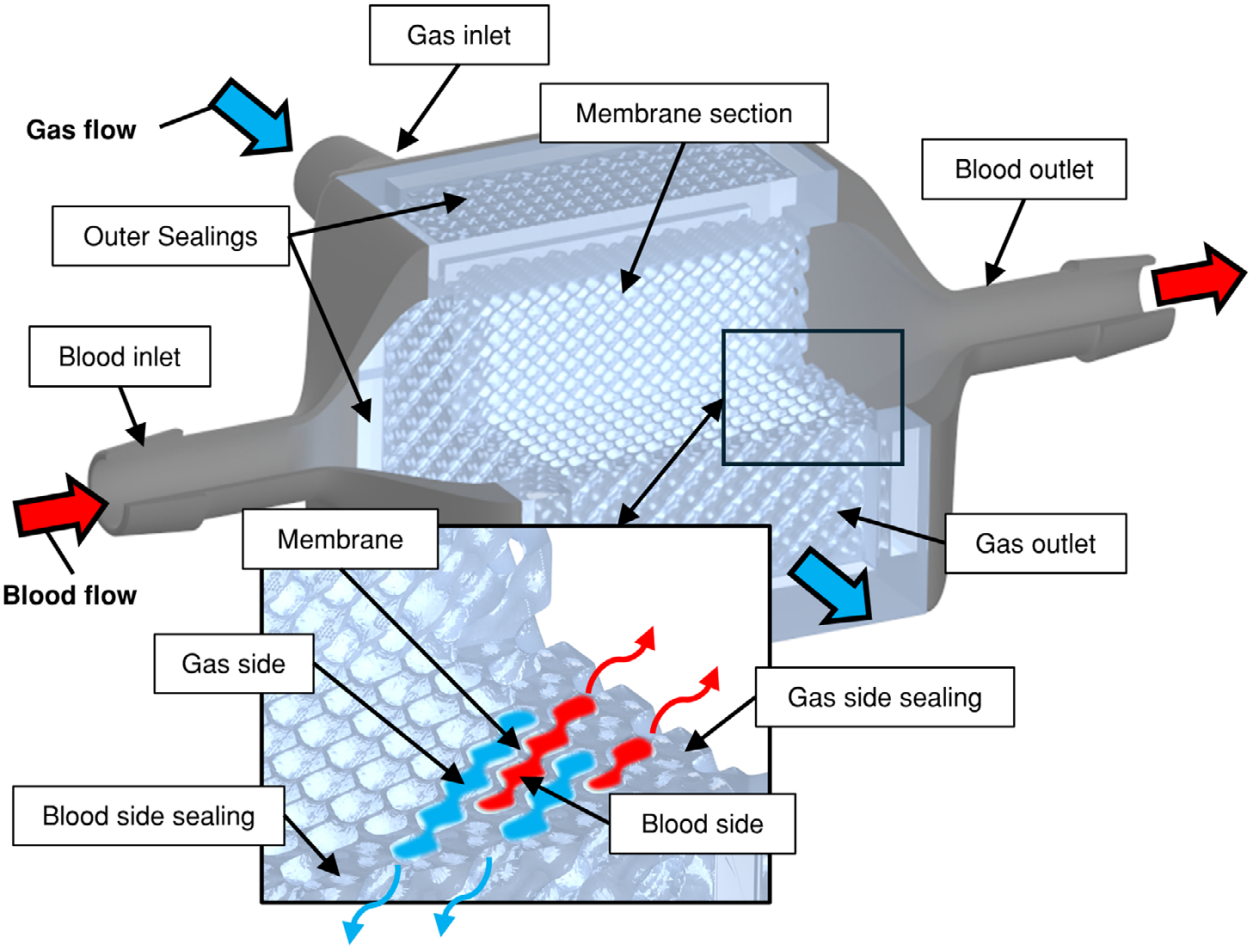
Rendering of the 3D membrane oxygenator prototype design.

A modular design was chosen for the 3D membrane oxygenator. This design consists of a membrane module and attached flow diverters that guide the blood and gas flow for an even distribution inside the membrane structure. Cubic geometries were defined as the building volume for the membrane module, allowing perpendicular flow of blood and sweep gas. The membrane separates the building volume into two regions through which blood and gas flow are guided. Each region is sealed outwardly on the sides of the modules, which are parallel to the respective fluid's flow direction (cf. **Figure** **7**; Figure S1, Supporting Information). This membrane module was produced using the manufacturing process described below. Three diffuser geometries were placed at the blood inlet and outlet, and the gas inlet. A Schwarz Diamond TPMS type was chosen for the membrane structure geometry. The tool geometry for the 3D printing was designed using a custom Python script (v3.10), making use of implicit geometry definitions. Here, the membrane geometry is defined by an implicit mathematical equation (cf. Equation 1).

(1) $$\cos\left(x\right)\cdot \cos\left(y\right)\cdot \cos\left(z\right)-\sin\left(x\right)\cdot \sin\left(y\right)\cdot \sin\left(z\right)=0$$

The script yielded an image stack in combination with g‐code instructions for geometry reconstruction, which was imported into Chitubox v2.1 (CBD Technology, Shenzhen, China) and resliced to create the proprietary file format for 3D printing, analogous to previously published work.^[^ ^38^ ^]^ The 3D printed tooling geometry corresponded to the volume of the blood flow channels in the final membrane module. Diffuser geometries were designed in Inventor Professional 2022 (Autodesk, San Francisco, USA) for the homogenous distribution of blood flow from one‐fourth″ tubing to the TPMS membrane module.

### Manufacturing

**Figure 8:**
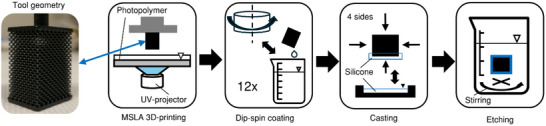
Process steps of the hybrid manufacturing approach for the 3D membrane modules.

The tool geometries were 3D printed on a Phrozen Sonic Mini 8K (Phrozen Tech, Hsinchu, Taiwan) masked stereolithography 3D printer. The printing resin (xMold, Nexa3D, Ventura, USA) was mixed with 2% (weight) black resin pigment (3DJake, Graz, Austria) for resolution fine‐tuning. Three‐dimensional printing was performed with a layer height of 20 µm and an exposure time of 1.8 s. Additional printing settings are provided in Table S4 (Supporting Information). After printing, the part was washed in fresh isopropanol for 20 min at a temperature of 50 °C for two cycles. Isopropanol was renewed between the cycles. Subsequently, the part was dried and then annealed in a vacuum oven at 160 °C for 2 h. Finally, the part was postcured in a high‐vacuum plasma oven (Diener electronic, Ebhausen, Germany) in a nitrogen atmosphere for 20 min. A siloxane compound (Elastosil RT620, Wacker Chemie, Munich, Germany) was chosen as the membrane material. First, the two compounds of the siloxane were hand‐mixed according to the manufacturer's recommendations. Afterward, the compound was diluted using n‐Heptane with a 1:1 weight ratio to reduce viscosity. The dip‐spin coating process consisted of the following steps that were repeated for each layer. First, the 3D printed part was slowly dipped into the diluted membrane material. Second, the part was mounted onto a centrifuge and spun at approximately 10 g. Lastly, the part was left in an oven at 50 °C for 30 min for layer curing. In total, twelve of these coating cycles were performed. The part was rotated after each coating cycle, changing the direction of acceleration inside the centrifuge. After coating, the sealing around the blood inlet and outlet sides and across the two adjacent outer surfaces was cast. Next, the 3D printed geometry was etched away in a 4% sodium hydroxide solution at 70 °C, ensuring good circulation of the solution with a magnetic stirrer. This etching process took approximately 90 h. After etching, the final membrane modules were first washed in citric acid for pH neutralization and then cleaned in isopropanol for 1 h. The manufacturing steps are provided in **Figure** **8**.

In total, six 3D membrane modules with a TPMS unit cell size of 3 mm were manufactured and assembled with the diffuser geometries. These diffuser parts were 3D printed on the same printer but with a different material (Phrozen aqua resin gray 8k, Phrozen Tech, Hsinchu, Taiwan). Three additional membrane modules with a TPMS unit cell size of 1 mm were manufactured using the same process steps but with eight coating layers. The HFM modules were manufactured using polymethylpentene (PMP) HFM mats (Oxyplus, Solventum, Wuppertal, Germany) with a configuration of 18 fibres per centimeter that were stacked in 90° orientation. They were integrated into a housing using the institute's standard for size variable HFM oxygenators.^[^ ^42^ ^]^ Manufacturing outcome of the 3D membrane was investigated using light‐ and field emission scanning electron microscopy. Therefore, manufacturing quality, shape accuracy, homogeneity, and surface structure were evaluated. Also, membrane thickness was measured using microscopic imaging at five different locations on three slice sections each in one prototype. Additionally, micro‐CT scans were performed with the 3D TPMS membrane modules (U‐CT, MILabs, Houten, Netherlands) using a voxel resolution of 160 µm. From the imaging data, measurements of blood flow channel dimensions were performed normal to the membrane walls at five arbitrary locations per prototype. Afterward, the minimum and maximum values were calculated as well as their average. Moreover, the active membrane surface area was identified based on the micro‐CT images as the volume with fully patent gas channels adjacent to the blood flow channels. For further calculation, an average membrane surface area was used for four of the modules, which all showed similar blockage of the gas channels (cf. Figure S1, Supporting Information). One of the TPMS modules featured a deviating surface area of 6911 mm^2^. Also, two HFM modules featured a diameter of 30 mm, a surface area of 37,973 mm^2^, and a length of 10 mm. Other prototypes were to the specifications in Table 1.

### Performance Testing

**Figure 9:**
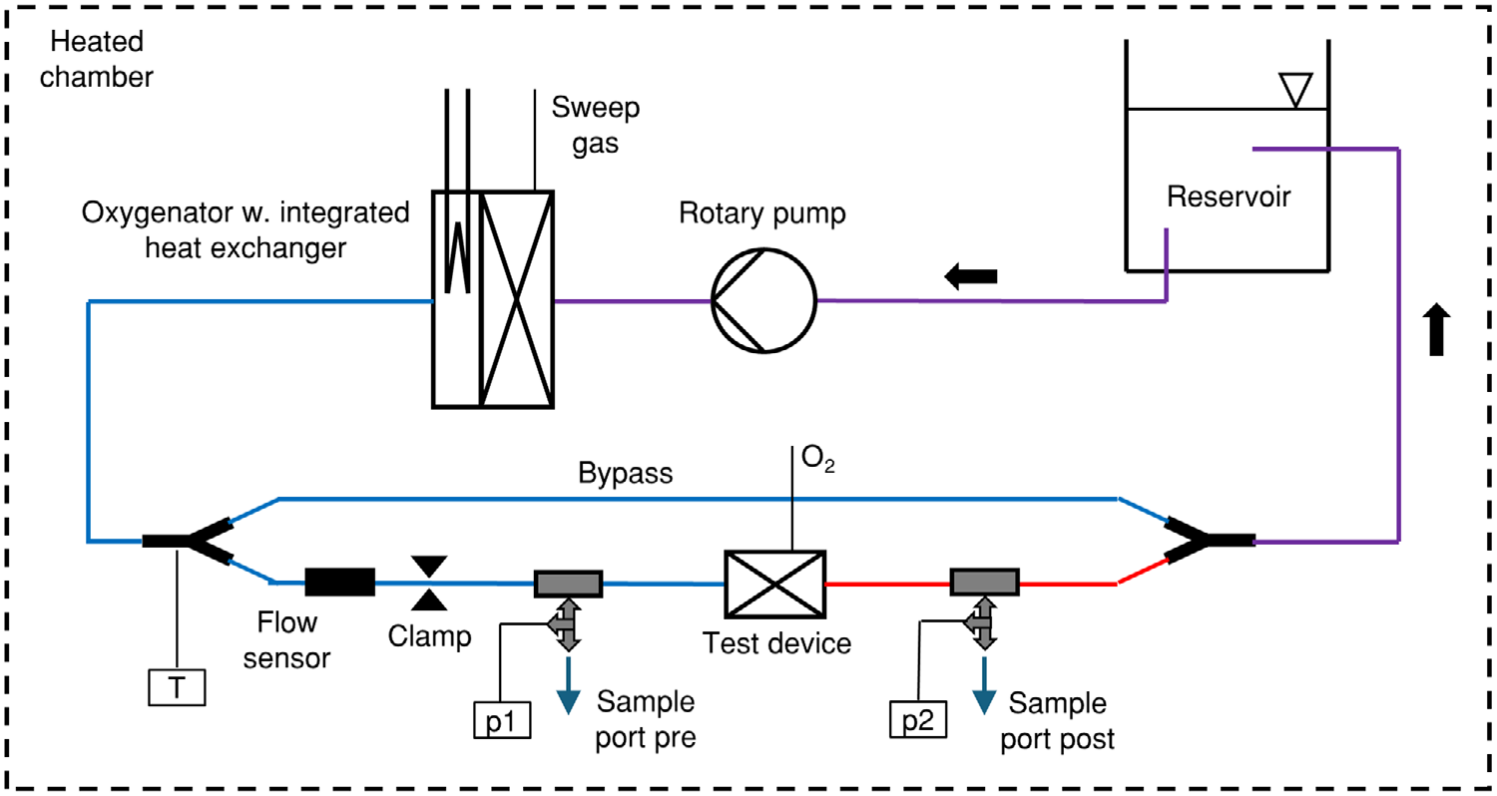
In vitro performance testing measuring setup. (T, temperature probe; p, pressure probe).

To ensure watertightness for the blood tests, all modules were primed with water, and 50 mmHg of pressure was applied. Performance testing according to ISO 7199 was performed with five prototypes for HFM and TPMS, each using porcine blood. The blood was fully heparinized with 15 000 IU per liter. Gas transfer rates and pressure drops were evaluated. The testing was performed using an in‐line circuit (cf. **Figure** **9**; Figure S7, Supporting Information), where venous blood gas values were established using a clinically used oxygenator (Quadrox‐i small adult, Maquet, Hechingen, Germany). This oxygenator was operated at an 800 mL min^−1^ blood flow rate and a sweep gas flow of 2 L min^−1^. Blood flow rates through the test specimens were established using a bypass and an adjustable resistance clamp. All measurements concerning the test specimens were performed at flow rates of 50, 100, and 150 mL min^−1^, which correspond to blood flow velocities and Reynolds numbers that are in the typical operating range of clinically used oxygenators.^[^ ^32^ ^]^ Sweep gas concentrations were adjusted to set venous blood gas concentrations according to the standard. The integrated heat exchanger within the oxygenator was used to set a blood temperature of 38 °C. A DP3 (Deltastream, Fresenius Medical Care, Germany) rotary pump was used to drive the circuit. Blood samples were taken at the inlet and outlets of the prototypes and analyzed using an ABL 800 (Radiometer Medical, Copenhagen, Denmark) device. Pressures were measured with Xtrans pressure transducers (Codan PVB Critical Care, Forstinning, Germany), and blood flow rates were metered using ultrasonic clamp‐on flow probes (ME6PXL, Transonic Systems, NY, USA). Pure oxygen was used as sweep gas. The sweep gas volume flow to blood flow ratio was set to a value of 10:1. Prior to blood contact, the circuit was primed with isotonic saline solution (0.9%), which was removed before filling the circuit with blood. In total, the circuit contained a volume of 1.5 L of blood.

Gas transfer rates were calculated from the gas concentration difference upstream and downstream of the prototypes and the blood flow rate (cf. Equation 2), where $V_{i}$ is the gas transfer rate, Q is the blood flow rate and *c*_i_ is the blood gas concentration, calculated by the blood gas analyzer. The index *i* denotes the gas species of oxygen or carbon dioxide.

(2) $$V_{i}=Q\cdot \left(c_{i,\mathrm{post}}-c_{i,\mathrm{pre}}\right)$$

Moreover, gas transfer coefficients, β, were calculated (cf. Equation 3) as well as length‐specific pressure drops (cf. Equation 5) and area goodness factors (Equation 6) as a dimensionless quantity of efficiency.

(3) $$\beta_{i}=\frac{V_{i}}{A\cdot \Delta c_{i,\log}}$$

(4) $$\Delta c_{i,\log}=\frac{c_{i,\mathrm{post}}-c_{i,\mathrm{pre}}}{\ln\left(\frac{c_{i,\mathrm{post}}-c_{i,\mathrm{ref}}}{c_{i,\mathrm{pre}}-c_{i,\mathrm{ref}}}\right)}$$

where *A* is the membrane surface area, and Δ*c*_i,log_ is the logarithmic mean concentration difference between the sweep gas and the blood inside the prototype.^[^ ^43^ ^]^ *c*_i,ref_ is a reference concentration of gas inside the blood with sweep gas partial pressures. Here, values of 0.18 mL_O2_, _n_ mL_blood_^−1^, and 0 mL_CO2, n_ mL_blood_^−1^ were used for oxygen and carbon dioxide, respectively.

(5) $$\Delta p_{\mathrm{spec}}=\frac{\Delta p}{L}$$

where Δ*p*_spec_ is the length‐specific pressure drop, Δ*p* is the measured pressure drop, and *L* is the length of the prototype's membrane section in flow direction. The overall gas transfer efficiency of the oxygenator prototypes was assessed using the area goodness factor *G*_a,i_, adapted for mass transfer, that determines a nondimensional ratio between gas transfer and pressure loss (cf. Equation 6).^[^ ^24^ ^]^

(6) $$G_{a,i}=\frac{Sh_{i}\cdot Re\cdot Sc_{i}^{-\frac{1}{3}}}{f}$$

(7) $$Sh_{i}=\frac{\beta_{i}\cdot L_{h}}{D_{i}}$$

(8) $$Sc_{i}=\frac{\eta }{\rho \cdot D_{i}}$$

(9) $$Re=\frac{\rho \cdot \bar{u}\cdot L_{h}}{\eta }$$

(10) $$f=\frac{\Delta p_{\mathrm{spec}}\cdot L_{h}}{2\cdot \rho \cdot {\bar{u}}^{2}}$$

where *Sh*_i_ is the Sherwood‐number, *Re* is the Reynolds‐number, *Sc*_i_ is the Schmidt‐number, *f* is the Fanning friction factor, *L*_h_ is the hydraulic reference dimension, *D*_i_ is the diffusion coefficient of the gas species inside blood^[^ ^44^, ^45^ ^]^ η is the blood viscosity, assumed to be a constant value of 3.4 mPa s, ρ is the blood density of 1050 kg m^−3^ and $\bar{u}$ is the average blood velocity inside the membrane modules. This velocity was calculated by dividing the blood flow rate by the cross‐sectional area and the membrane structure volume porosity (0.5 for the 3D structure and 0.46 for HFM). For carbon dioxide, a constant diffusion coefficient of 7.36e^−10^ m^2^ s^−1^ was used.^[^ ^45^ ^]^ To account for the effect of the chemical binding of oxygen to hemoglobin inside the erythrocytes, the diffusion coefficient for oxygen was calculated depending on the derivative of the oxygen dissociation curve.^[^ ^46^ ^]^

(11) $$D_{O_{2}}=\frac{D_{O_{2},b}}{1+\frac{c_{\mathrm{Hb}}}{\alpha_{O_{2},b}}\cdot \frac{\partial S_{O_{2}}}{\partial P_{O_{2}}}}$$

where $D_{O_{2}}$ is the diffusivity of oxygen in blood of 1.8e^−9^ m^2^ s^−1^, *c*_Hb_ is the volumetric hemoglobin binding capacity of blood being 0.167, $\alpha_{O_{2},b}$ is the solubility of oxygen in blood being 2.64e^−7^ Pa^−1^ and $\frac{\partial S_{O_{2}}}{\partial P_{O_{2}}}$ is the derivative of the oxygen dissociation curve with respect to oxygen partial pressure.^[^ ^45^, ^46^ ^]^ In addition to the values calculated from the measurement data, an empirical fit of the Sherwood‐number (cf. Equation 12) was used for a prediction of area goodness factors over a larger range of Reynolds‐numbers in combination with a linear fit of specific pressure drops.^[^ ^23^ ^]^

(12) $$Sh_{i}=a\cdot Re^{b}\cdot Sc_{i}^{\frac{1}{3}}$$

When corrected for human blood, the measured oxygen saturation values were adjusted according to the measured mean differences between human and porcine blood, depending on oxygen partial pressure, which were reported by Serianni et al.^[^ ^28^ ^]^ Measurements of the membrane material's permeability for oxygen and carbon dioxide were performed in accordance with ISO 15 105 and in comparison to Sylgard 184 (Dow Chemical Company, Michigan, USA), which is commonly used in MAL.^[^ ^17^, ^47^ ^]^ Moreover, two samples of the membrane material were measured. One without treatment and the other one after 90 h submerging in sodium hydroxide solution at 70 °C, like in the etching process for membrane module manufacturing, followed by washing in isopropanol. Diffusion coefficients of the different samples were evaluated from the permeability measurement data using the time‐lag method.^[^ ^48^ ^]^ Blood flow and mass transfer inside small sections of TPMS membrane structures were simulated using our previously reported approach.^[^ ^32^ ^]^ Here, the oxygen and carbon dioxide transfer coefficients, determined from a 110 µm membrane thickness and the measured diffusivities of the membrane material after etching and cleaning, were implemented as boundary conditions. Area goodness factors of 1, 2, and 3 mm TPMS unit cell sizes were calculated identically to the experimental results (cf. Figure S4, Supporting Information).

Additionally, computational fluid dynamics simulations were performed for the HFM structure and the 1 and 3 mm TPMS unit cell size structures using a previously reported approach.^[^ ^32^ ^]^ From the simulation results, shear spatial shear stress contours on the membrane wall were evaluated as well as histograms of shear stress magnitude at a 150 mL min^−1^ flow rate (cf. Figure S5, Supporting Information). Maximum operating pressure was investigated with one of the five TPMS membrane modules by slowly increasing the blood side pressure using water and taking microscopic images of the structure from the gas outlet (cf. Figure S6, Supporting Information).

### Statistical Analysis

Statistical analysis was performed in MATLAB R2023b (MathWorks, Natick, USA). Normal distribution was verified using a Shapiro–Wilk test. Equal variances were investigated using an *f*‐test. Significant differences in measurement data (gas transfer coefficient and specific pressure drop) between the two prototypes were evaluated using Welch's *t*‐test. A *p*‐value was considered significant with *p* < 0.05.

## Conflict of Interest

The authors declare no conflict of interest.

## Supporting information



Supporting Information

## Data Availability

The data that support the findings of this study are available from the corresponding author upon reasonable request.
