# Supplementary material for: 3D Membrane Microstructures for Increased Efficiency in Blood‐Gas Transfer
Source: Adv Sci (Weinh). 2025 Nov 7;12(47):e12302. doi: 10.1002/advs.202512302 (PMC12713010; doi:10.1002/advs.202512302)
Supplement: Supplementary file 1 — Supporting Information [file ADVS-12-e12302-s001.docx]

Supporting Information

3D Membrane Microstructures for Increased Efficiency in Blood-Gas Transfer

*Kai P. Barbian*, F. Neuhaus, L. T. Hirschwald, J. Linkhorst, M. Wessling, B. Wiegmann,
 J. M. Focke, U. Steinseifer, M. Neidlin and S. V. Jansen*


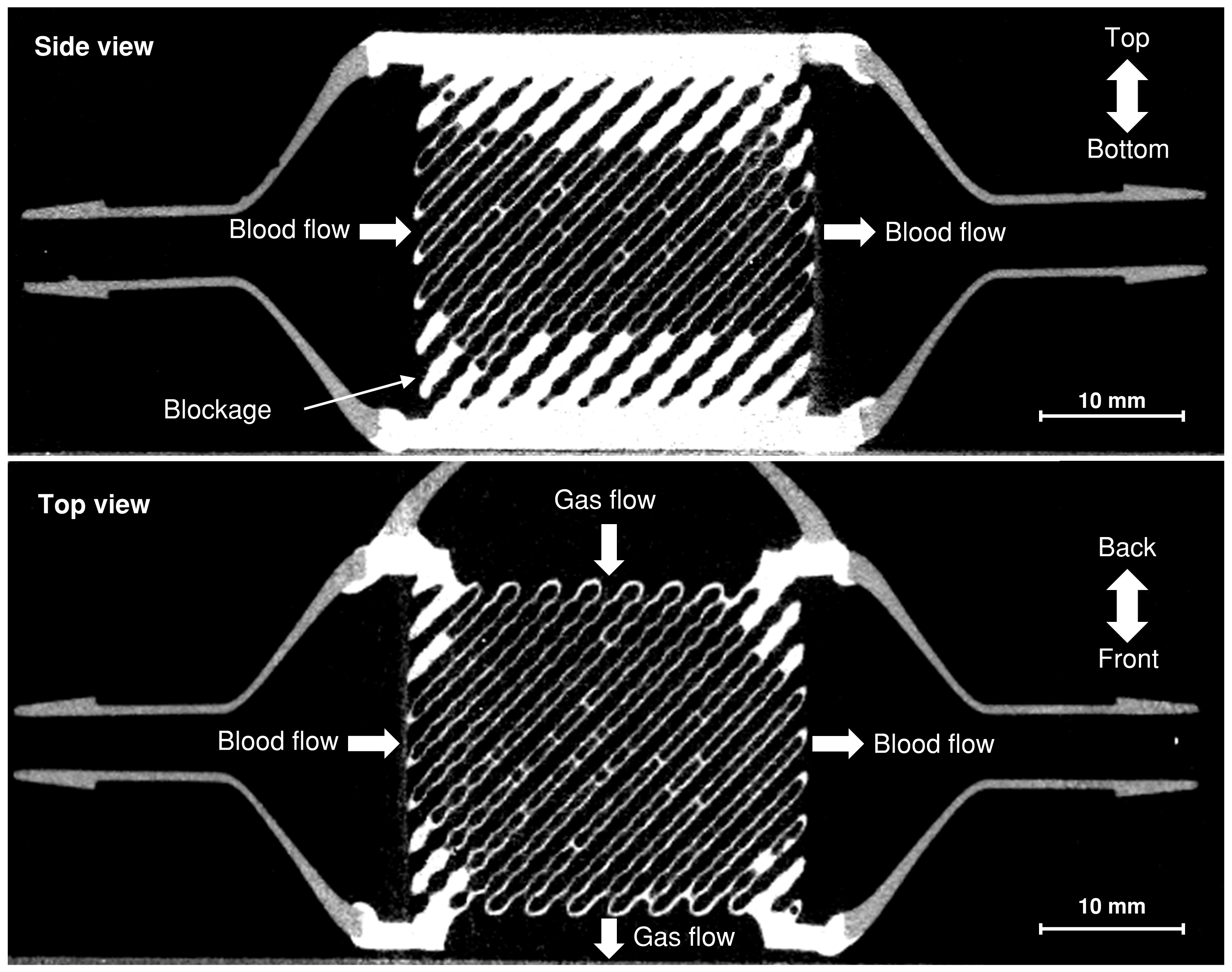


Figure S1: Micro-CT images of TPMS module with visible occlusion of parts of the gas channels that were introduced during the sealing castings. No blockages were observed on the blood side.


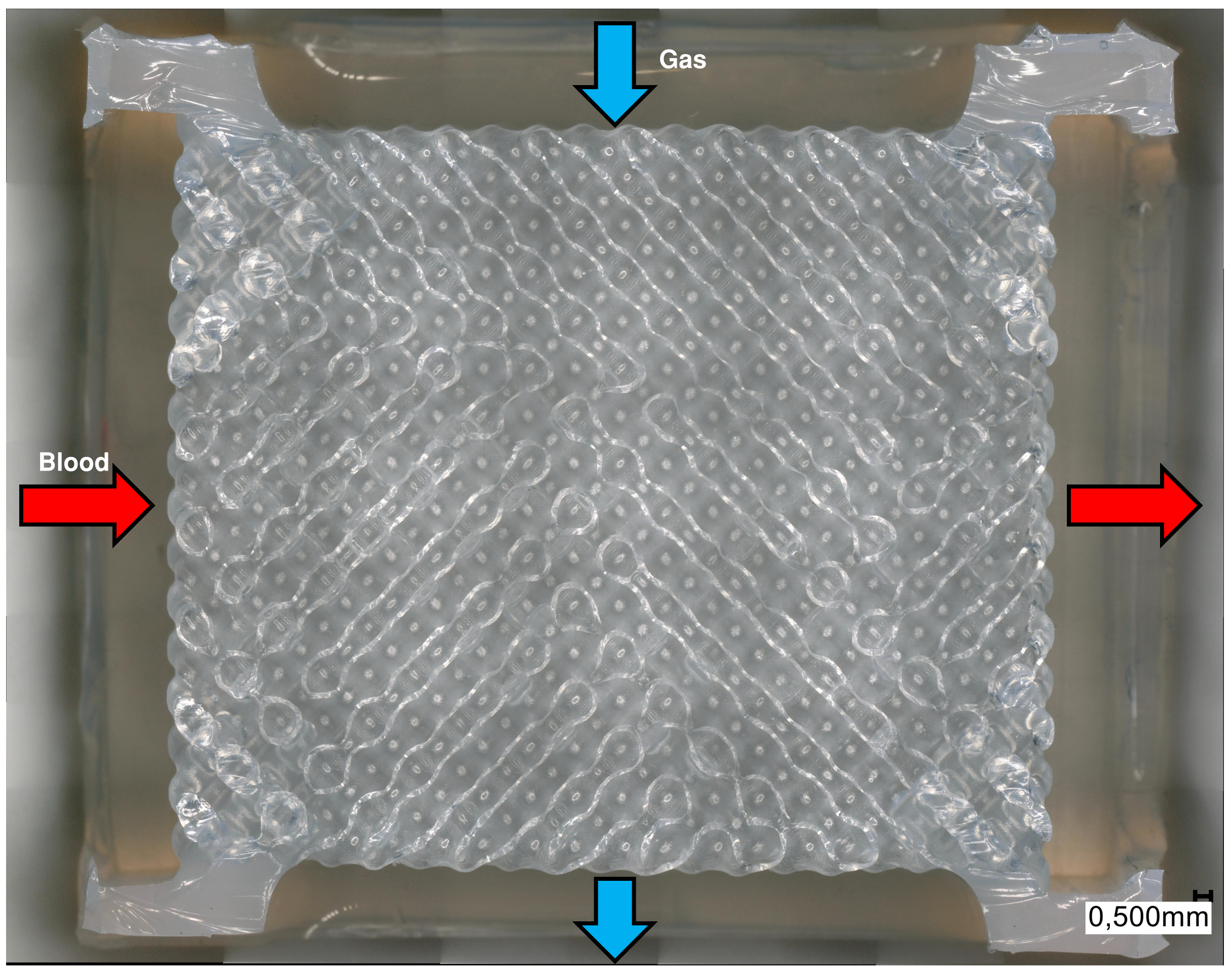


Figure S2: Microscope view of full cross section cut through the center of a TPMS oxygenator module showing homogenous membrane thickness distribution.


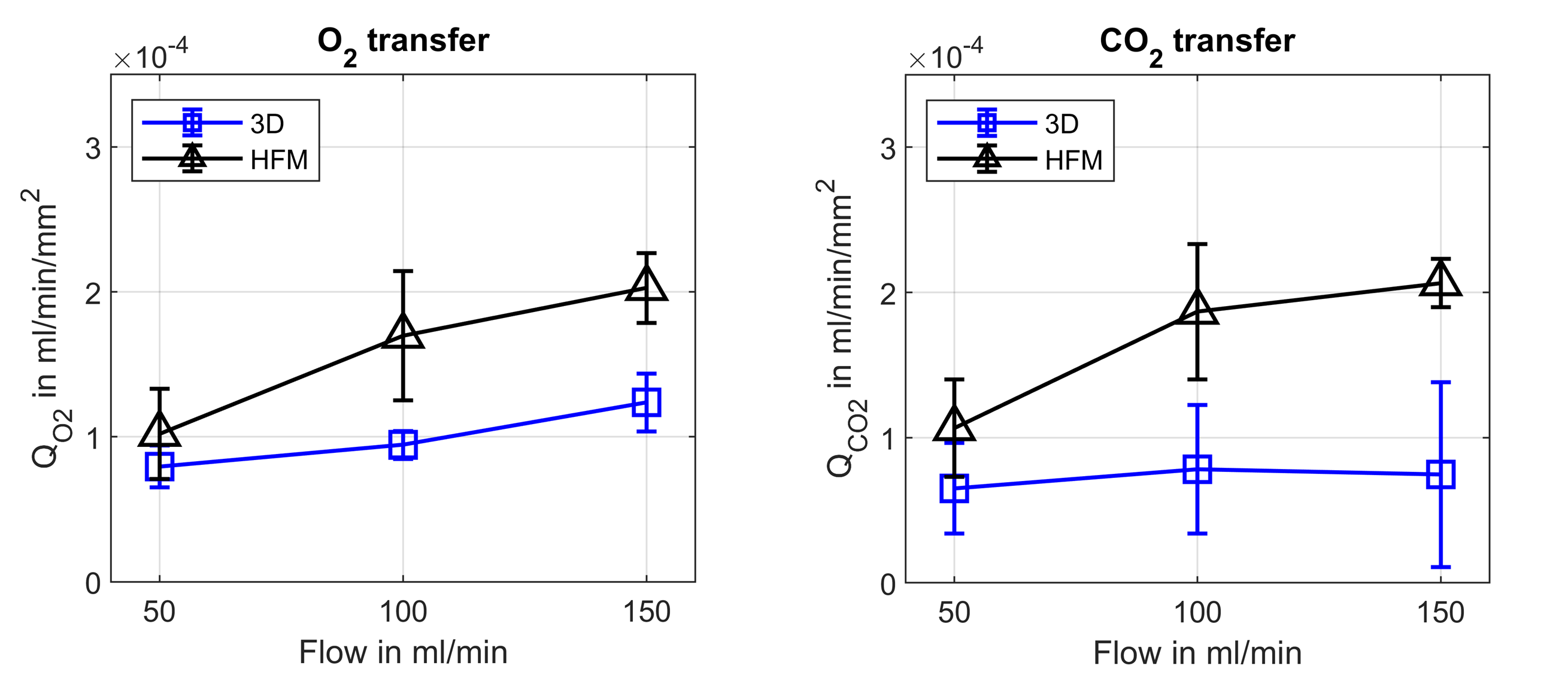


Figure S3: Area specific gas transfer performance for 3D membrane structure (“3D”, blue line with square markers) and HFM (“HFM”, black line with triangle markers) of oxygen (left) and carbon dioxide (right), presented as mean values and standard deviation for n=5 prototypes.


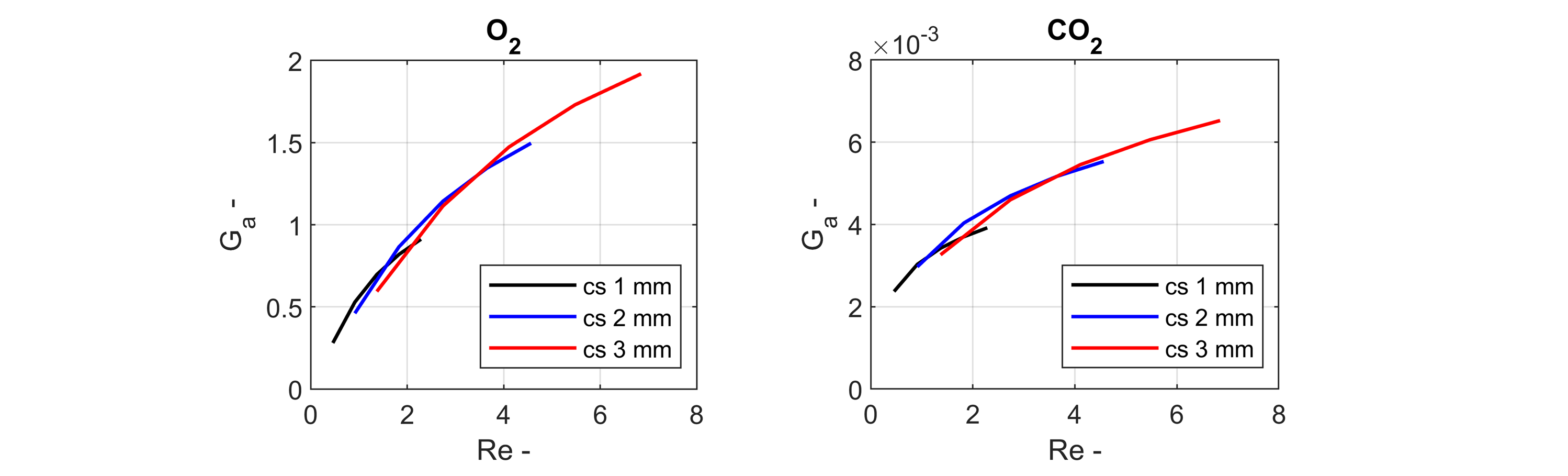


Figure S4: Area goodness factors (G_a_) for oxygen and carbon dioxide transfer over Reynolds-number for 1 mm (“cs 1 mm”, black line), 2 mm (“cs 2 mm”, blue line) and 3 mm (“cs 3 mm”, red line) TPMS unit cells, calculated from numerical simulations of flow and mass transfer in TPMS structures*. Inside the investigated range of clinically relevant flow velocities, the G_a_ curves of different TPMS unit cell sizes show a consistent progression with a maximum deviation of less than 12% between each other for oxygen and carbon dioxide.


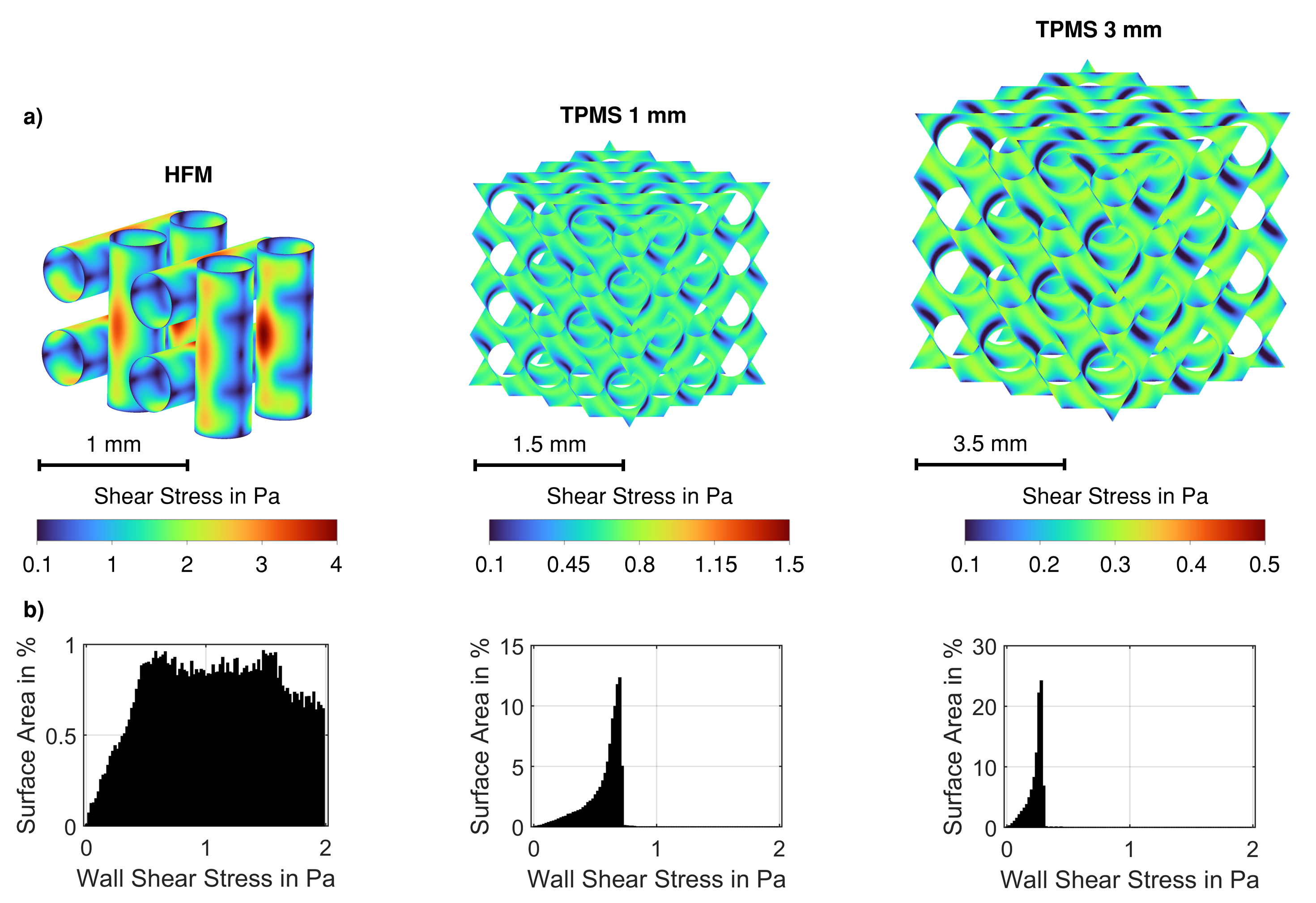


Figure S5: Shear Stress distribution in 2x2x2 periodical unit elements of HFM and TPMS membrane structures corresponding to the prototype configurations at 150 ml min^-1^. a) Wall shear stress contour on HFM (left), TPMS membrane with 1 mm unit cell size (center) and TPMS membrane with 3 mm unit cell size (right). b) Histograms of shear stress values on HFM (left), TPMS membrane with 1 mm unit cell size (center) and TPMS membrane with 3 mm unit cell size (right). Shear stress contours and distributions are calculated using the same, previously reported model*.

*Barbian, K. P. *et al.* Flow and mass transfer prediction in anisotropic TPMS-structures as extracorporeal oxygenator membranes using reduced order modeling. *Journal of Membrane Science* **690,** 122160; 10.1016/j.memsci.2023.122160 (2024).


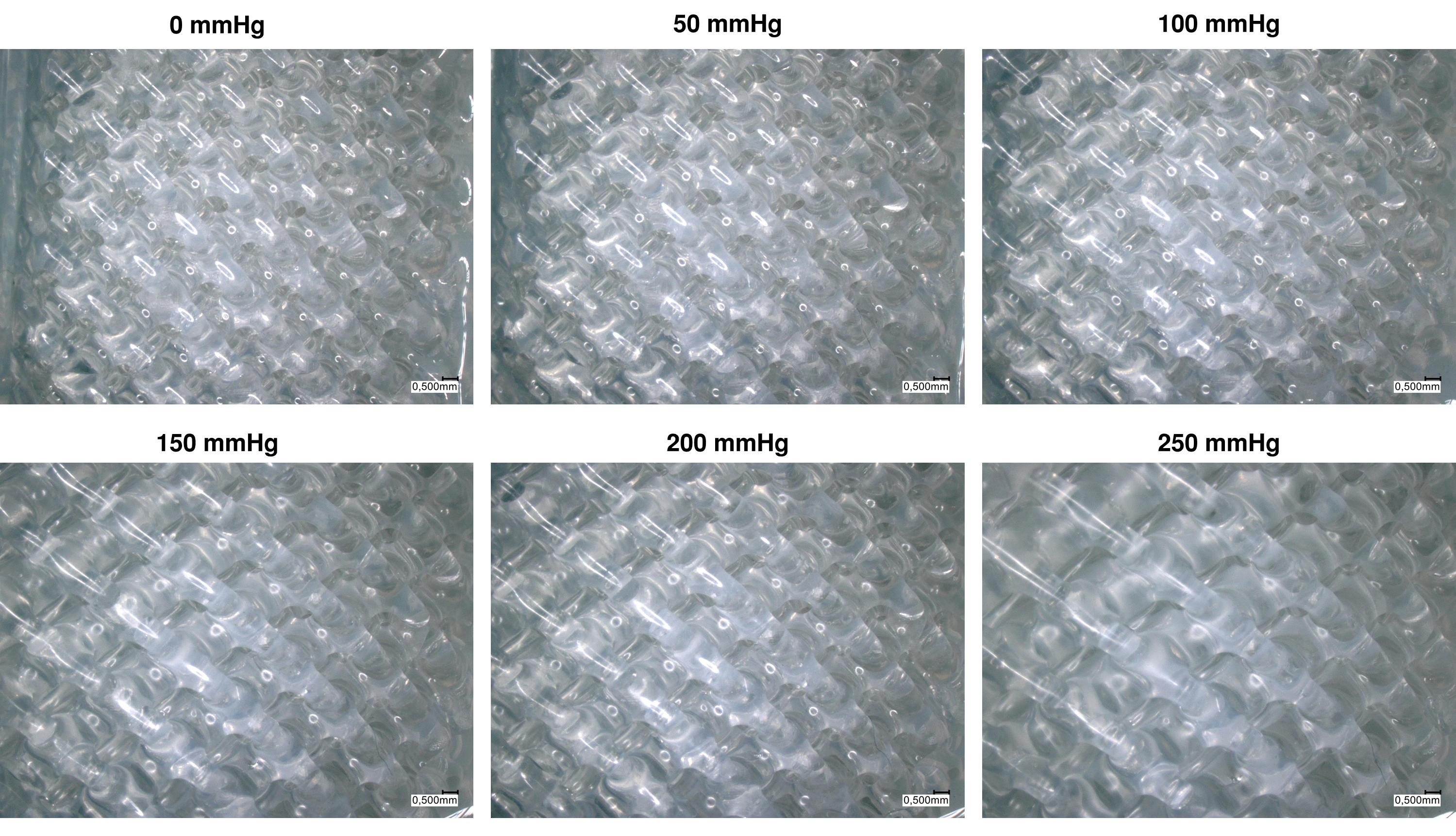


Figure S6: Microscopic images of gas outlet of TPMS oxygenator module with different pressure loads on blood side. Values are relative to atmospheric pressure.


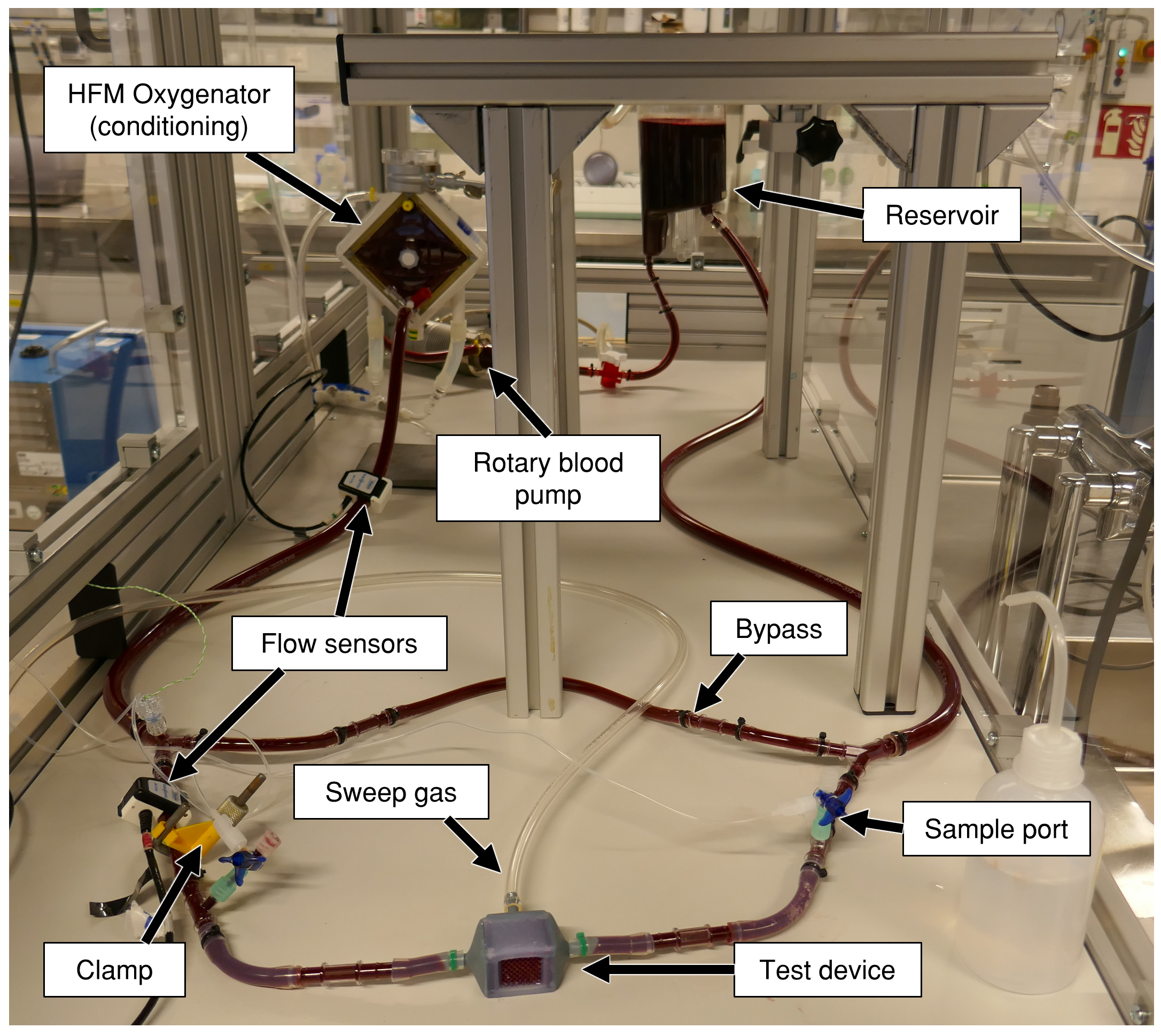


Figure S7: Image of experimental setup for gas transfer and pressure drop measurements with TPMS oxygenator module.

Table S1: Raw data of blood sample measurements during gas transfer testing

| **ID** | **Membrane surface area in mm²** | **Flow rate in ml min^-1^** | **Loca-tion** | **Hb in g dl^-1^** | **Hkt in %** | **pH** | **p_O2_ in mmHg** | **sO2 in %** | **c_O2_ in ml_O2_ dl_blood_^-1^** | **p_CO2_ in mmHg** | **c_CO2_ in ml_O2_ dl_blood_^-1^** | **HCO3^-^ in mmol l^-1^** | **O2 transfer in ml min^-1^ m^-2^** | **CO2 transfer in ml min^-1^ m^-2^** |
| --- | --- | --- | --- | --- | --- | --- | --- | --- | --- | --- | --- | --- | --- | --- |
| 3D #1 | 8294.1 | 50 | inlet | 12.9 | 39.6 | 7.28 | 46.3 | 65.3 | 11.5 | 45.9 | 43.8 | 21.1 | 60.28 | -84.40 |
|  |  |  | outlet | 12.7 | 39 | 7.29 | 52.9 | 72.2 | 12.5 | 43.7 | 42.4 | 20.4 |  |  |
|  |  | 100 | inlet | 12.8 | 39.5 | 7.28 | 47 | 65.2 | 11.4 | 46 | 44 | 21.1 | 96.45 | -60.28 |
|  |  |  | outlet | 12.8 | 39.2 | 7.29 | 49.4 | 70 | 12.2 | 45.2 | 43.5 | 20.9 |  |  |
|  |  | 150 | inlet | 12.9 | 39.6 | 7.29 | 46 | 65.5 | 11.5 | 46.4 | 44.3 | 21.3 | 90.43 | -54.26 |
|  |  |  | outlet | 12.8 | 39.5 | 7.29 | 47.9 | 68.8 | 12 | 46 | 44 | 21.2 |  |  |
| 3D #2 | 8294.1 | 50 | inlet | 12.8 | 39.3 | 7.28 | 45.7 | 65.1 | 11.3 | 45.5 | 42.7 | 20.4 | 96.45 | -72.34 |
|  |  |  | outlet | 12.6 | 38.9 | 7.28 | 52.1 | 75.1 | 12.9 | 43.9 | 41.5 | 19.9 |  |  |
|  |  | 100 | inlet | 12.8 | 39.2 | 7.27 | 46.5 | 66.1 | 11.5 | 46.5 | 43.3 | 20.7 | 108.51 | -96.45 |
|  |  |  | outlet | 12.7 | 39 | 7.28 | 49.6 | 71.7 | 12.4 | 45.4 | 42.5 | 20.4 |  |  |
|  |  | 150 | inlet | 12.8 | 39.3 | 7.27 | 47.1 | 66.9 | 11.7 | 46.2 | 42.8 | 20.5 | 126.60 | -54.26 |
|  |  |  | outlet | 12.8 | 39.3 | 7.27 | 49.7 | 71.2 | 12.4 | 45.7 | 42.5 | 20.4 |  |  |
| 3D #3 | 8294.1 | 50 | inlet | 12.6 | 38.7 | 7.34 | 46.3 | 70.1 | 12 | 47.6 | 51.6 | 25.1 | 90.43 | -102.48 |
|  |  |  | outlet | 12.5 | 38.4 | 7.35 | 52.3 | 79.8 | 13.5 | 45.1 | 49.9 | 24.4 |  |  |
|  |  | 100 | inlet | 12.6 | 38.7 | 7.34 | 44 | 65.9 | 11.3 | 46.6 | 50.6 | 24.6 | 84.40 | -60.28 |
|  |  |  | outlet | 12.5 | 38.6 | 7.35 | 45.4 | 70.4 | 12 | 45.9 | 50.1 | 24.4 |  |  |
|  |  | 150 | inlet | 12.6 | 38.8 | 7.34 | 43.6 | 65.2 | 11.2 | 47 | 50.9 | 24.8 | 126.60 | -180.85 |
|  |  |  | outlet | 12.6 | 38.7 | 7.34 | 45.1 | 69.4 | 11.9 | 45.8 | 49.9 | 24.3 |  |  |
| 3D #4 | 8294.1 | 50 | inlet | 12.5 | 38.4 | 7.31 | 43.5 | 65.4 | 11.1 | 46.6 | 47 | 22.6 | 78.37 | -30.14 |
|  |  |  | outlet | 12.4 | 38.2 | 7.31 | 49.5 | 73.6 | 12.4 | 45.7 | 46.5 | 22.5 |  |  |
|  |  | 100 | inlet | 12.4 | 38.2 | 7.31 | 43.6 | 65.5 | 11.1 | 48 | 48.2 | 23.2 | 96.45 | -144.68 |
|  |  |  | outlet | 12.4 | 38.1 | 7.31 | 46.6 | 70.3 | 11.9 | 46.7 | 47 | 22.6 |  |  |
|  |  | 150 | inlet | 12.5 | 38.3 | 7.31 | 43.4 | 66.6 | 11.3 | 46.8 | 47.1 | 22.7 | 144.68 | -72.34 |
|  |  |  | outlet | 12.5 | 38.3 | 7.31 | 46.9 | 71 | 12.1 | 46.3 | 46.7 | 22.5 |  |  |
| 3D #5 | 6911.7 | 50 | inlet | 12.4 | 38.2 | 7.39 | 46.3 | 68.1 | 11.8 | 42.8 | 52 | 24.4 | 72.34 | -36.17 |
|  |  |  | outlet | 12.4 | 38.1 | 7.40 | 50.5 | 73.7 | 12.8 | 42.1 | 51.5 | 24.7 |  |  |
|  |  | 100 | inlet | 12.4 | 38.1 | 7.39 | 45.7 | 68.3 | 11.9 | 42.5 | 51.6 | 24.6 | 86.81 | -28.94 |
|  |  |  | outlet | 12.4 | 38.1 | 7.39 | 48.7 | 71.9 | 12.5 | 42.3 | 51.4 | 24.5 |  |  |
|  |  | 150 | inlet | 12.4 | 38.3 | 7.39 | 44.3 | 68.2 | 11.9 | 42.2 | 51.2 | 24.7 | 130.21 | -10.85 |
|  |  |  | outlet | 12.4 | 381 | 7.40 | 47.3 | 71.9 | 12.5 | 41.9 | 51.15 | 24.7 |  |  |

| **ID** | **Membrane surface area in mm²** | **Flow rate in ml min^-1^** | **Loca-tion** | **Hb in g dl^-1^** | **Hkt in %** | **pH** | **p_O2_ in mmHg** | **sO2 in %** | **c_O2_ in ml_O2_ dl_blood_^-1^** | **p_CO2_ in mmHg** | **c_CO2_ in ml_O2_ dl_blood_^-1^** | **HCO3^-^ in mmol l^-1^** | **O2 transfer in ml min^-1^ m^-2^** | **CO2 transfer in ml min^-1^ m^-2^** |
| --- | --- | --- | --- | --- | --- | --- | --- | --- | --- | --- | --- | --- | --- | --- |
| HFM #1 | 15822.2 | 50 | inlet | 12.6 | 38.7 | 7.33 | 43.9 | 66.1 | 11.3 | 47.5 | 50.5 | 24.5 | 126.40 | -151.69 |
|  |  |  | outlet | 12.4 | 38.2 | 7.38 | 58.5 | 91.1 | 15.3 | 39.3 | 45.7 | 22.5 |  |  |
|  |  | 100 | inlet | 12.5 | 38.5 | 7.33 | 43.7 | 68.1 | 11.6 | 46.6 | 49.5 | 24 | 202.25 | -208.57 |
|  |  |  | outlet | 12.6 | 38.8 | 7.35 | 57.2 | 86.6 | 14.8 | 41.8 | 46.2 | 22.7 |  |  |
|  |  | 150 | inlet | 12.6 | 38.6 | 7.33 | 43.3 | 67.3 | 11.5 | 46.5 | 49.2 | 23.9 | 237.01 | -218.05 |
|  |  |  | outlet | 12.6 | 38.6 | 7.35 | 53.3 | 82 | 14 | 42.5 | 46.9 | 23 |  |  |
| HFM #2 | 15822.2 | 50 | inlet | 12.6 | 38.8 | 7.33 | 40.8 | 63.8 | 11 | 45.8 | 48 | 23.3 | 129.56 | -126.40 |
|  |  |  | outlet | 12.5 | 38.5 | 7.37 | 59.7 | 88.8 | 15.1 | 38.2 | 44 | 21.7 |  |  |
|  |  | 100 | inlet | 12.6 | 38.8 | 7.32 | 43.3 | 65.7 | 11.3 | 47.7 | 49.5 | 23.9 | 208.57 | -240.17 |
|  |  |  | outlet | 12.5 | 38.3 | 7.35 | 54.4 | 86.6 | 14.6 | 41.8 | 45.7 | 22.3 |  |  |
|  |  | 150 | inlet | 12.6 | 38.7 | 7.32 | 43.8 | 67.1 | 11.5 | 47.1 | 48.9 | 23.7 | 208.57 | -208.57 |
|  |  |  | outlet | 12.5 | 38.6 | 7.34 | 51.9 | 80.6 | 13.7 | 43.3 | 46.7 | 22.8 |  |  |
| HFM #3 | 15822.2 | 50 | inlet | 12.6 | 38.6 | 7.32 | 42.7 | 65.4 | 11.2 | 46.2 | 47.6 | 23 | 113.76 | -82.16 |
|  |  |  | outlet | 12.5 | 38.4 | 7.35 | 56.4 | 87.3 | 14.8 | 41.1 | 45 | 22 |  |  |
|  |  | 100 | inlet | 12.6 | 38.6 | 7.32 | 44.7 | 66.9 | 11.5 | 47 | 48.4 | 23.4 | 195.93 | -208.57 |
|  |  |  | outlet | 12.5 | 38.5 | 7.35 | 57.6 | 86.1 | 14.6 | 41.3 | 45.1 | 22.1 |  |  |
|  |  | 150 | inlet | 12.6 | 38.6 | 7.32 | 45.1 | 67.1 | 11.5 | 46.9 | 48.1 | 23.2 | 208.57 | -218.05 |
|  |  |  | outlet | 12.5 | 38.5 | 7.33 | 54.6 | 81 | 13.7 | 43.4 | 45.8 | 22.3 |  |  |
| HFM #4 | 37973 | 50 | inlet | 12.7 | 38.9 | 7.31 | 41.5 | 61.1 | 10.8 | 45.3 | 46.2 | 22.3 | 84.27 | -104.02 |
|  |  |  | outlet | 12.6 | 38.8 | 7.39 | 87.6 | 97.2 | 17.2 | 32.3 | 38.3 | 19 |  |  |
|  |  | 100 | inlet | 12.7 | 39.2 | 7.31 | 43.4 | 64.3 | 11.5 | 45.5 | 46.2 | 22.3 | 123.77 | -147.47 |
|  |  |  | outlet | 12.7 | 39.2 | 7.37 | 73.6 | 90.5 | 16.2 | 35.6 | 40.6 | 20.1 |  |  |
|  |  | 150 | inlet | 12.7 | 39 | 7.31 | 44.3 | 65.2 | 11.6 | 45.7 | 46.4 | 22.4 | 177.76 | -209.36 |
|  |  |  | outlet | 12.7 | 38.9 | 7.35 | 71 | 90.6 | 16.1 | 37.6 | 41.1 | 20.2 |  |  |
| HFM #5 | 37973 | 50 | inlet | 12.7 | 39.1 | 7.29 | 42.1 | 61.5 | 11 | 46.5 | 45.5 | 21.9 | 56.62 | -68.47 |
|  |  |  | outlet | 12.6 | 38.9 | 7.34 | 63 | 86.4 | 15.3 | 37.6 | 40.3 | 19.7 |  |  |
|  |  | 100 | inlet | 12.7 | 39 | 7.30 | 42.9 | 63 | 11.2 | 46 | 45.1 | 21.7 | 118.51 | -129.04 |
|  |  |  | outlet | 12.7 | 38.9 | 7.34 | 68.7 | 88.7 | 15.7 | 37.3 | 40.2 | 19.7 |  |  |
|  |  | 150 | inlet | 12.7 | 39.2 | 7.30 | 44.2 | 65 | 11.6 | 45 | 44.2 | 21.3 | 181.71 | -177.76 |
|  |  |  | outlet | 12.7 | 38.9 | 7.35 | 72.9 | 91.5 | 16.2 | 36.2 | 39.7 | 19.5 |  |  |

Table S2: Raw data from pressure drop measurement during blood testing

| **ID** | **Module length in mm** | **Flow rate in ml min^-1^** | **p1 in mmHg** | **p2 in mmHg** | **dp in mmHg** | **dp_spec_ in mmHg mm^-1^** |
| --- | --- | --- | --- | --- | --- | --- |
| 3D #1 | 28 | 0 (calibration) | 32.2 | 33.9 | -1.7 | - |
|  |  | 50 | 33 | 34.2 | 0.5 | 0.018 |
|  |  | 100 | 33.8 | 34.4 | 1.1 | 0.039 |
|  |  | 150 | 34.4 | 34.6 | 1.5 | 0.054 |
|  |  | 200 | 35 | 34.8 | 1.9 | 0.068 |
|  |  | 250 | 35.7 | 35 | 2.4 | 0.086 |
| 3D #2 | 28 | 0 (calibration) | 31.3 | 32.8 | -1.5 | - |
|  |  | 50 | 33.1 | 33.9 | 0.7 | 0.025 |
|  |  | 100 | 33.9 | 34.2 | 1.2 | 0.043 |
|  |  | 150 | 34.5 | 34.5 | 1.5 | 0.054 |
|  |  | 200 | 35 | 34.6 | 1.9 | 0.068 |
|  |  | 250 | 35.8 | 34.7 | 2.6 | 0.093 |
| 3D #3 | 28 | 0 (calibration) | 33 | 33.9 | -0.9 | - |
|  |  | 50 | 33.5 | 34 | 0.4 | 0.014 |
|  |  | 100 | 34 | 34.2 | 0.7 | 0.025 |
|  |  | 150 | 34.6 | 34.4 | 1.1 | 0.039 |
|  |  | 200 | 35.3 | 34.6 | 1.6 | 0.057 |
|  |  | 250 | 36 | 34.7 | 2.2 | 0.079 |
| 3D #4 | 28 | 0 (calibration) | 30.7 | 32.1 | -1.4 | - |
|  |  | 50 | 32.5 | 33.4 | 0.5 | 0.018 |
|  |  | 100 | 33 | 33.6 | 0.8 | 0.029 |
|  |  | 150 | 33.7 | 33.8 | 1.3 | 0.046 |
|  |  | 200 | 34.3 | 33.9 | 1.8 | 0.064 |
|  |  | 250 | 35 | 34.1 | 2.3 | 0.082 |
| 3D #5 | 28 | 0 (calibration) | 32.9 | 34.5 | -1.6 | - |
|  |  | 50 | 32.1 | 33.2 | 0.5 | 0.018 |
|  |  | 100 | 32.7 | 33.4 | 0.9 | 0.032 |
|  |  | 150 | 33.2 | 33.5 | 1.3 | 0.046 |
|  |  | 200 | 33.8 | 33.6 | 1.8 | 0.064 |
|  |  | 250 | 34.6 | 33.8 | 2.4 | 0.086 |

| **ID** | **Module length in mm** | **Flow rate in ml min^-1^** | **p1 in mmHg** | **p2 in mmHg** | **dp in mmHg** | **dp_spec_ in mmHg mm^-1^** |
| --- | --- | --- | --- | --- | --- | --- |
| HFM #1 | 6 | 0 (calibration) | 42.5 | 43.5 | -1 | - |
|  |  | 50 | 34.3 | 33.8 | 1.5 | 0.250 |
|  |  | 100 | 35.8 | 34 | 2.8 | 0.467 |
|  |  | 150 | 37.7 | 34.2 | 4.5 | 0.750 |
|  |  | 200 | 39.8 | 34.4 | 6.4 | 1.067 |
|  |  | 250 | 43 | 35.4 | 8.6 | 1.433 |
| HFM #2 | 6 | 0 (calibration) | 27.2 | 28 | -0.8 | - |
|  |  | 50 | 34.2 | 33.6 | 1.4 | 0.233 |
|  |  | 100 | 35.8 | 33.8 | 2.8 | 0.467 |
|  |  | 150 | 37.5 | 34 | 4.3 | 0.717 |
|  |  | 200 | 40.2 | 34.5 | 6.5 | 1.083 |
|  |  | 250 | 43.2 | 35.4 | 8.6 | 1.433 |
| HFM #3 | 6 | 0 (calibration) | 32.1 | 32.9 | -0.8 | - |
|  |  | 50 | 34.4 | 33.5 | 1.7 | 0.283 |
|  |  | 100 | 35.8 | 33.7 | 2.9 | 0.483 |
|  |  | 150 | 38.1 | 33.9 | 5 | 0.833 |
|  |  | 200 | 40.9 | 34.5 | 7.2 | 1.200 |
|  |  | 250 | 44.4 | 35.5 | 9.7 | 1.617 |
| HFM #4 | 10 | 0 (calibration) | 0 | 0 | 0 | - |
|  |  | 50 | 31 | 28 | 3 | 0.300 |
|  |  | 100 | 33 | 28 | 5 | 0.500 |
|  |  | 150 | 35 | 28 | 7 | 0.700 |
|  |  | 200 | 40 | 29 | 11 | 1.100 |
|  |  | 250 | 44 | 31 | 13 | 1.300 |
| HFM #5 | 10 | 0 (calibration) | 0 | 0 | 0 | - |
|  |  | 50 | 30 | 27 | 3 | 0.300 |
|  |  | 100 | 33 | 27 | 6 | 0.600 |
|  |  | 150 | 37 | 28 | 9 | 0.900 |
|  |  | 200 | 41 | 30 | 11 | 1.100 |
|  |  | 250 | 45 | 31 | 14 | 1.400 |

Table S3: Results of permeability and diffusivity measurements of the membrane material Elastosil RT620 before (a) and after (b) etching and cleaning in comparison to Sylgard 184.

| **Specimen** | **Oxygen** | | **Carbon dioxide** | |
| --- | --- | --- | --- | --- |
|  | **Permeability in  1e-14 m^2^ s^-1^ Pa^-1^** | **Diffusivity in 1e-9 m^2^ s^-1^** | **Permeability in 1e-13 m^2^ s^-1^ Pa^-1^** | **Diffusivity in  1e-9 m^2^ s^-1^** |
| RT 620 (a) | 1.56 | 1.13 | 1.29 | 2.84 |
| RT 620 (b) | 2.17 | 1.43 | 1.08 | 2.52 |
| Sylgard 184 | 2.23 | 1.22 | 1.54 | 2.73 |

Table S4: 3D printing settings for the manufacturing of the sacrificial tool geometry.

| **Setting** | **Value** |
| --- | --- |
| Layer height | 20 µm |
| Pixel size in µm | 24 µm x 24 µm |
| Bottom layer count | 6 |
| Bottom exposure time | 24 s |
| Transition | Linear, 6 layers |
| Rest time before lift | 0.5 s |
| Rest time after retract | 3 s |
| Exposure time | 1.8 s |
